# Naturally occurring asbestos and its possible environmental and health implications in the Basilicata region (Southern Italy)

**DOI:** 10.1007/s10653-026-03314-5

**Published:** 2026-06-25

**Authors:** Andrea Bloise, Paolo Ballirano, Ilaria Fuoco, Maria Cristina Di Carlo, Carmine Apollaro, Giovanni Vespasiano, Marco Paccapelo, Lorenzo Arrizza, Melinda Darby Dyar, Elizabeth Sklute, Alessandra Altieri, Antonella Campopiano, Annapaola Cannizzaro, Maria Rosaria Bruno, Giacomo Malvasi, Alessandro Pacella

**Affiliations:** 1 Department of Biology, Ecology and Earth Sciences, University of Calabria, 87036 Arcavacata di Rende, CS Italy; 2 Department of Earth Sciences, Sapienza University of Rome, Piazzale Aldo Moro 5, 00185 Rome, Italy; 3 Planetary Science Institute, 1700 East Fort Lowell, Suite 106, Tucson, AZ USA; 4 Department of Medicine, Epidemiology, Occupational and Environmental Hygiene, National Institute for Insurance Against Accidents at Work (INAIL), Rome, Italy; 5 Radioactivity and Asbestos Office ARPA Basilicata, Potenza, Italy

**Keywords:** Asbestos, Fibrous tremolite, Naturally occurring asbestos, Basilicata region

## Abstract

Asbestos that occurs in the environment and has not been extracted for commercial purposes is commonly referred to as Naturally Occurring Asbestos (NOA). Research has shown a higher-than-expected incidence of mesothelioma in populations residing near NOA sites across California, Greece, Turkey, Cyprus, Corsica, and New Caledonia. In Italy, in the area around Pollino Massif (Basilicata region), a total of 124 mesothelioma cases were documented among population living in the villages of Castelluccio Superiore and Inferiore, Lauria, Latronico, Episcopia, San Severino Lucano, and Francavilla in Sinni. In this work for the first time, we report the morphological and chemical structural characterization of asbestos tremolite samples from the NOA outcrops in the Pollino area (Basilicata region). The detailed sample characterization has been attained by using a multi-analytical approach (EMP, SEM-EDS, TEM-EDS, Mössabuer, X-ray powder diffraction, and thermal analysis). Morphological investigation highlighted that a significant fraction of each sample (ca. 50%) consists of long fibres (> 5 µm) having width below 0.25 µm, considered the most carcinogenic fibres. Additionally, all asbestos samples showed the presence of Fe at the octahedral sites of the tremolite structure. This is relevant from the toxicological point of view because the presence of Fe in the fibre structure is a critical factor in asbestos pathogenicity. Obtained results provide a solid foundation for better understanding the potential health risks associated with asbestos tremolite in the Pollino Massif area. They also highlight the importance of constant monitoring and risk assessment to protect local communities from asbestos exposure.

**Supplementary Information:**

The online version contains supplementary material available at 10.1007/s10653-026-03314-5.

## Introduction

The term “asbestos” is a commercial designation for six fibrous minerals, which are grouped into two main categories: serpentine (chrysotile) and amphiboles (including asbestos tremolite, asbestos actinolite, asbestos anthophyllite, amosite, and crocidolite) (WHO, 1986). Asbestos is classified by the IARC as a Group 1 carcinogen (IARC, 2012) due to its ability to cause various respiratory diseases upon inhalation (Addison & McConnell, 2008; Lippmann, 2014). Its toxicity and pathogenicity result from the combined influence of factors such as morphology, surface reactivity, and biopersistence (Berry et al., 2022; Bloise et al., 2020a, 2024; Pacella et al., 2018, 2020). Although the use and commercialization of asbestos have been banned in many countries, environmental exposure continues to pose a serious concern (Gualtieri, 2023).

Asbestos that occurs in the environment, that have not been extracted for commercial purposes, is commonly referred to as Naturally Occurring Asbestos (NOA). It is typically found in ophiolitic complexes as a result of natural geological processes (e.g., Bloise et al., 2021; Harper, 2008; Pacella et al., 2010, 2021; Ross & Nolan, 2003). In Italy, these complexes occur in the Alps and Apennines (Apollaro et al., 2019, 2021; Cavallo, 2020; Fuoco et al., 2020; Ricchiuti et al., 2020; Vignaroli et al., 2014), with well-documented asbestos-bearing serpentine and amphibole veins in regions such as Liguria, Piedmont, Latium (Ballirano et al., 2008; Cavallo, 2020; Pacella et al., 2010; Vignaroli et al., 2014), Calabria, and Basilicata (Bloise et al., 2014, 2017a, 2020b; Dichicco et al., 2019).

Research has shown a higher-than-expected incidence of mesothelioma in populations residing near NOA sites across California, Greece, Turkey, Cyprus, Corsica, and New Caledonia (e.g., Baumann et al., 2015; Langer, 2008; Luce et al., 2000; Ross & Nolan, 2003). In response, several U.S. states such as Virginia, Maryland, Pennsylvania, and California have introduced asbestos exposure control measures specifically targeting construction activities (Lee et al., 2008; Ross & Nolan, 2003). In regions where NOA is present, fibres may be dispersed into air, soil, and water as a result of natural processes like weathering or through human activities (Bloise et al., 2016; Petriglieri et al., 2023), creating a potential health hazard. Tremolite crystals are frequently encountered in a range of geological settings (Bloise et al., 2017a, 2020a, 2020b, 2021; Pereira et al., 2025), and their disturbance can lead to the release of respirable fibres (length > 5 µm, width < 3, aspect ratio ≥ 3:1, measured by scanning electron and phase contrast microscopy) (Gualtieri, 2017; Pacella et al., 2024; WHO, 1997). Recent investigations in Basilicata’s Pollino area have revealed a high incidence of pleural mesothelioma, asbestosis, and other lung diseases potentially linked to environmental exposure to tremolite asbestos (Bernardini et al., 2003; Burragato et al., 2007, 2010; Caputo et al., 2018; Musti et al., 2006; Pasetto et al., 2004). A total of 124 mesothelioma cases were documented among population living in the villages of Castelluccio Superiore and Inferiore, Lauria, Latronico, Episcopia, San Severino Lucano, and Francavilla in Sinni (a total of over 11,934 people) from 1993 to 2021, with pleural mesothelioma accounting for 91.9% and peritoneal mesothelioma for 8.1% (INAIL, 2024). Additionally, the Pollino area contains numerous abandoned serpentinite quarries that were historically exploited for aggregate extraction used in walls, railway ballast, flooring, roads, and general construction materials (Beneduce et al., 2008, 2012).

In a recent study, Pacella et al. (2024) report that tremolite from the Iacolinei quarry, in the town of Castelluccio Superiore (Basilicata Region), consists of long fibres that readily split into thin, respirable fibrils, with more than half measuring under 0.25 μm in diameter, a size associated with high carcinogenic potential (Wylie & Korchevskiy, 2023). Consequently, populations living in villages around the Pollino massif are today considered at high risk of asbestos exposure.

In this context, for the first time, the present study focuses on a detailed morphological and chemical structural characterization of asbestos tremolite samples from the NOA outcrops in the Pollino area (Basilicata region), employing a well-tested multi-analytical approach: scanning and transmission electron microscopies combined with energy dispersive spectrometry (SEM-EDS and TEM-EDS), Electron Microprobe (EMP), Mössbauer spectroscopy, X-ray powder diffraction (XRPD), thermal analysis (TG-DSC). Obtained results provide a solid foundation for better understanding the potential health risks associated with asbestos tremolite in this area. They also highlight the importance of continued monitoring and risk assessment to protect local communities from asbestos exposure.

### Geological setting

**Fig. 1:**
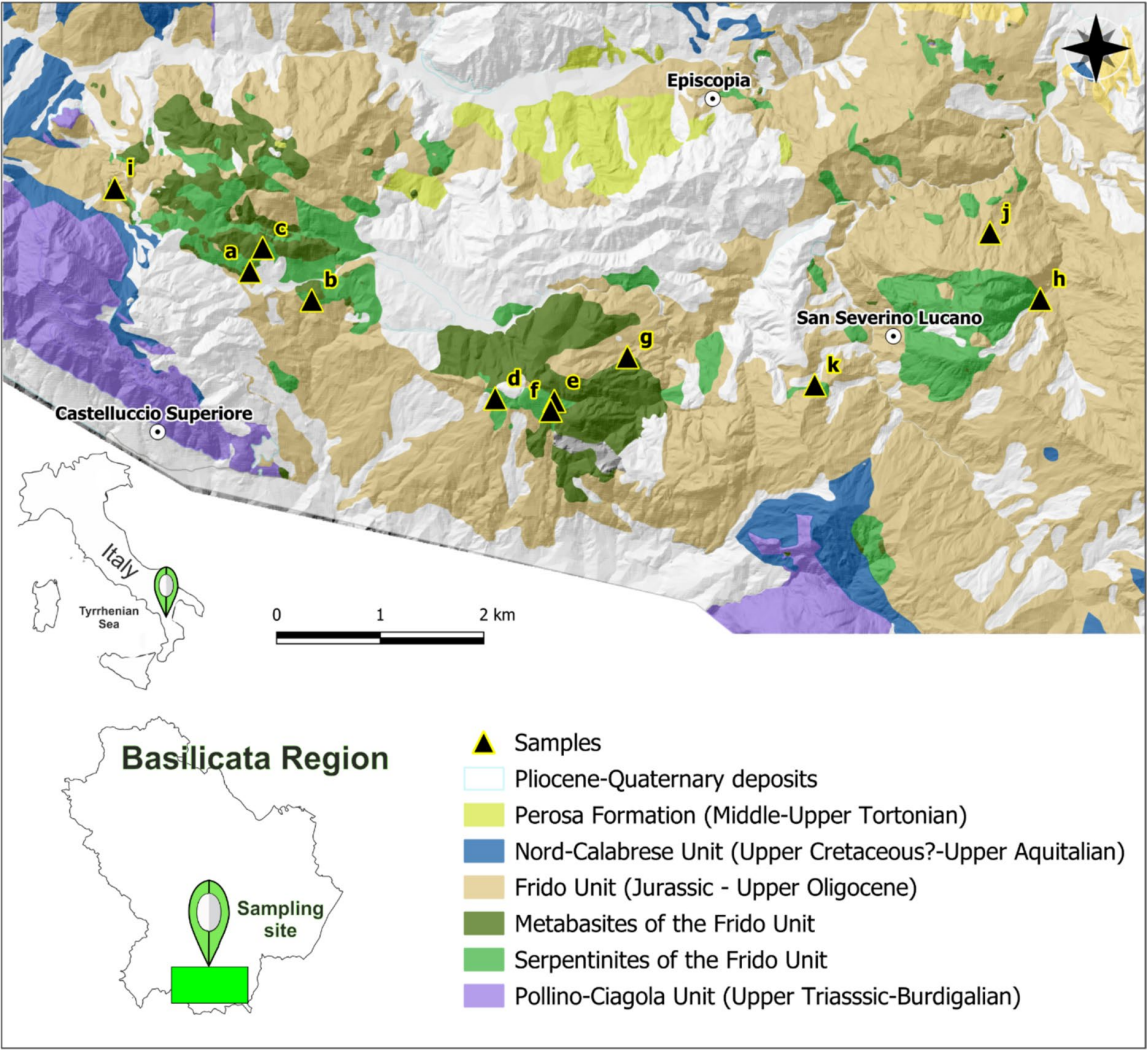
Simplified geological map of the Pollino Massif and location of the samples (modified from Vitale et al., 2013). **a** Perruttiere; **b** Serrapollo; **c** Radicata; **d** Serra Fagosa; **e** Pastoroso; **f** Pastoroso Pidocchioso; **g** Destra di Cornaleta; **h** Cava Timpa Castello; **i** Tempa Bruciata; **j** Sagittario; **k** Falascoso

The investigated area is located in the Basilicata region (southern Italy), along the northern slope of the Pollino Massif (Fig. 1). This sector of the southern Apennines is structurally complex and shaped by a combination of compressional and extensional tectonics linked to the Neogene–Quaternary evolution of the region (Giano et al., 2017; Guagliardi et al., 2016; Schiattarella et al., 1998). The regional deformation produced a nappe stack composed of east-verging thrusts, subsequently dissected by normal and strike-slip faults associated with the Pollino tectonic domain. The dominant structural grain trends NW–SE, in line with the orientation of the main ridges and valleys. Active fault systems, including the Pollino, Rotonda, Laino, and Mercure faults, have contributed to a mild extensional regime and shaped the present-day morphology (Papanikolaou et al., 2022). Geologically, the Pollino Massif consists of a series of thrust belts derived from the deformation of the African passive margin, active between the Oligocene and the Pleistocene. This tectonic evolution involved the ophiolitic lithosphere of the Ligurian Ocean (Bonardi et al., 2001), represented in this area by the Ligurian Complex. The complex includes two ophiolite-bearing units: the non-metamorphic Calabro–Lucanian Flysch Unit and the metamorphic Frido Unit, both of which experienced intense deformation (Critelli & Le Pera, 2009). The Frido Unit, extensively exposed in the study area, forms the uppermost tectonic element of the Ligurian accretionary complex (Monaco & Tortorici, 1991) and tectonically overlies the carbonate platform units of the Apennines (Pollino-Ciagola Unit). Lithologically, it is classically subdivided into two sub-units: the lower consisting of clayey schists with metarenites, metasiltites, quartzites, and metalimestones; the upper composed of limeschists, marbles, and intercalated greenish quartzites and pelitic layers. Within the Frido Unit, ophiolitic bodies occur as lenticular masses embedded in the metasedimentary succession. These include serpentinized peridotites and metabasites, often affected by intense fracturing and hydrothermal alteration. The serpentinites are related to metamorphosed ophiolitic sequences, where mafic magmatic dykes intrude medium- to high-grade metamorphic rocks (Sansone & Rizzo, 2012). These ophiolite bodies underwent two phases of ocean floor metamorphism (Sansone & Rizzo, 2012), during which ductile deformation, recrystallization, and hydrothermal metasomatism led to the formation of the current mineral assemblage, including fibrous minerals. The serpentinites have been classified as either cataclastic or massive in texture (Dichicco et al., 2015). Both types show similar mineralogy, dominated by serpentine, tremolite–actinolite, chlorite, magnetite, and Cr-bearing spinels, with minor amounts of calcite, dolomite, and clay minerals (Bloise et al., 2017a). Numerous cross-cutting fractures are observed, frequently filled with fibrous minerals that can be easily mobilized by surface runoff and weathering.

#### Sampling and characterization

**Fig. 2:**
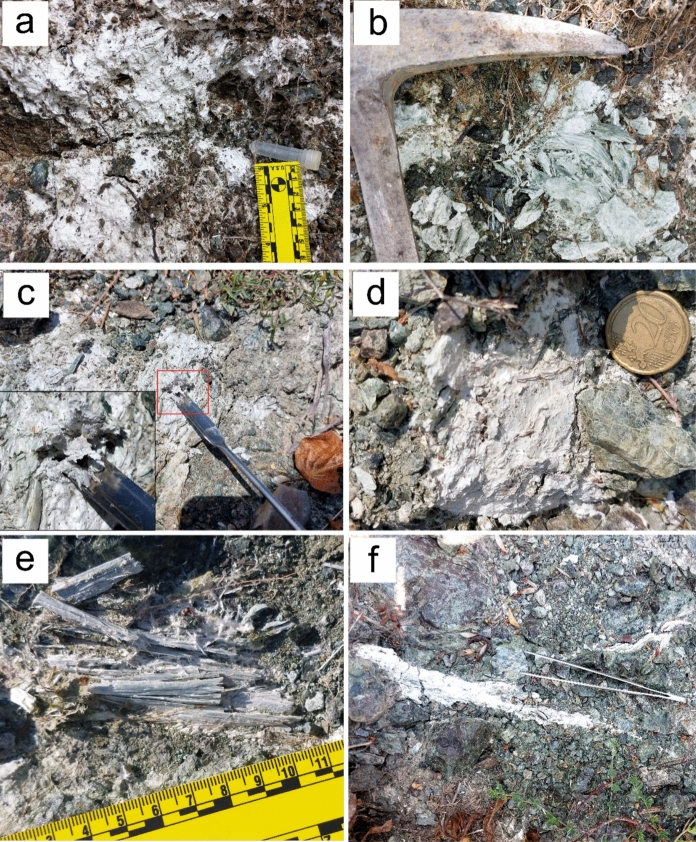

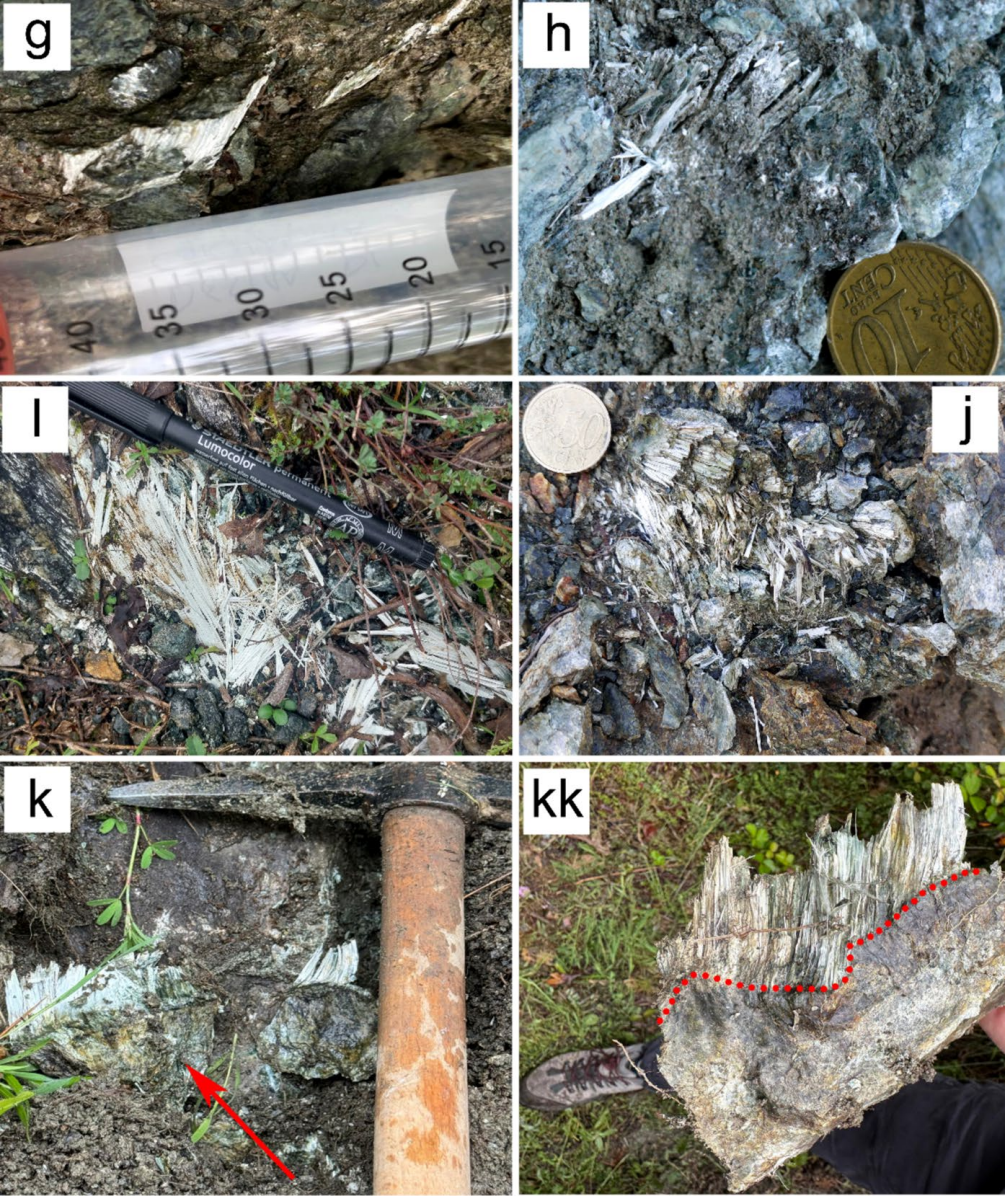
Field photograph at the sampling sites: **a** Perruttiere;** b** Serrapollo; **c** Radicata; **d** Serra Fagosa; **e** Pastoroso; **f** Pastoroso Pidocchioso; **g** Destra di Cornaleta;** h** Cava Timpa Castello;** i** Tempa Bruciata;** j** Sagittario;** k** Falascoso (the red arrow indicates the tremolite fibers (shown in detail in figure kk)

The study area, where the investigated NOA-bearing sequences crop out, includes several villages (Table 1; Fig. 1). Eleven tremolite asbestos samples were collected from various disturbed serpentinite outcrops in areas with regular human activity (farming, sports). Sampling sites included a housing construction site, unpaved road, landslide slope, excavation front, and inactive quarry (Supplementary Fig. S1), all contexts where natural fibre release is enhanced by mechanical disturbance. The serpentinite is generally exposed, with sparse vegetation cover, making it highly susceptible to weathering. Field observations revealed that tremolite asbestos occurs as white fibres in various settings, including within mineralized veins, dispersed throughout the surrounding rock matrix, as remnants of rock alteration, and in the soil, where it appears either as partially disaggregated nodules or as fully disaggregated fibrous powder (Fig. 2).

**Table 1:** Location, coordinates and geological site of sampling

Municipality	Site area	ID	UTM E	UTM N	Geological site
Castelluccio superiore	Perruttiere	**a**	584226,72	4432521,31	Excavation for house Construction
Castelluccio inferiore	Serrapollo	**b**	585420,56	4431964,24	Road cutting
Castelluccio superiore	Radicata	**c**	584473,00	4432975,00	Outcrop
Castelluccio superiore	Serra Fagosa	**d**	588964,50	4430065,80	Dirt road
Viggianello	Pastoroso	**e**	590106,00	4430018,00	Excavation front
Viggianello	Pastoroso Pidocchioso	**f**	590037,00	4429832,00	Outcrop
Viggianello	Destra di Cornaleta	**g**	591518,00	4430867,00	Landslide
Chiaromonte	Cava Timpa Castello	**h**	599493,00	4431985,00	Inactive quarry
Lauria	Tempa Bruciata	**i**	581616,05	4434121,61	Landslide slope
Chiaromonte	Sagittario	**j**	598520,36	4433264,83	Excavation front
Viggianello	Falascoso	**k**	595135,37	4430326,95	Excavation front

## Materials and methods

### Scanning electron microscope (SEM)

A Field-Emission Scanning Electron Microscope with Gemini electron optics column ZEISS Crossbeam 350, equipped with EDS (Octane Elite Plus) microanalysis, was employed to ascertain the morphology of fibres and fibre bundles, with a view to undertaking preliminary chemical analysis. For SEM investigations, a fragment of each sample was fixed onto an SEM stub using double-sided conductive adhesive tape and subsequently coated with graphite using a Quorum Q150T ES sputter coater.

### Transmission electron microscopy (TEM)

For the determination of size and structural features of the tremolite fibres, we employed a Jeol JEM 1400 Plus Transmission Electron Microscope (TEM) operating at 120 kV, equipped with Jeol large-area silicon drift detector SDD-EDS for microanalyses. For TEM investigation the sample was immersed in isopropyl alcohol and sonicated. No grinding or other mechanical particle-size reduction was performed. Subsequently, three drops of the resulting suspension were deposited onto a Formvar carbon-coated copper grid. The measured fibre widths therefore refer to fibres naturally present in the fine fraction of the sample, detached from larger aggregates only by sonication. To describe the size of tremolite, 60 TEM micrographs for each sample were captured, and 200 single fibers were measured. Measurements of tremolite fibres were conducted using OPTIKA PROView image analysis software.

### Thermogravimetric analysis

The samples were analyzed for weight changes and energetic transformations using simultaneous Thermogravimetric Analysis and Differential Scanning Calorimetry (TG-DSC) on a Netzsch STA 449 C Jupiter instrument. For thermal analysis, approximately 20 mg of finely ground sample material was used for each run. Sample underwent heating at a rate of 10 °C min^−1^ within the temperature range of 25–1200 °C under an air flow of 30 mL min^−1^. Derivative thermogravimetry (DTG) and exo- and endothermic peaks were analyzed using Netzsch Proteus thermal analysis software. Instrumental precision was verified through three repeated collections on a kaolinite reference sample, demonstrating good reproducibility (instrumental theoretical temperature precision of ± 1.2 °C), with a DSC detection limit < 1 µW and theoretical mass sensitivity of 0.10 µg.

### Electron microprobe (EMP)

The chemical composition of the tremolite fibres was determined using a JEOL JXA-iSP100 Super Probe equipped with five wavelength-dispersive spectrometers using the following conditions: excitation voltage 15 kV, specimen current 15 nA, beam diameter 5 µm, 20 s count time (peak), except for Si and Na (10 s), 10 s count time (background). The following standards were used: quartz (Si K_α_), ilmenite (Ti K_α_), chromite (Fe K_α_), metallic Mn (Mn K_α_), diopside (Mg K_α_), wollastonite (Ca K_α_), orthoclase (Al and K K_α_), albite (Na K_α_), metallic Cr (Cr K_α_), fluorapatite (F K_α_), and sylvite (Cl K_α_).

### X-ray powder diffraction

X-ray powder diffraction data were measured with a D8 Advance automated powder diffractometer (Bruker AXS, Karlsruhe, Germany) running in θ/θ transmission mode on capillaries. The instrument is equipped with an incident beam focusing graded multilayer Göbel mirror and a PSD VÅntec-1. The sample was gently ground in an agate mortar under ethanol and the resulting powder was loaded in a 0.6 mm diameter borosilicate glass capillary. The diffraction pattern was collected in step-scan mode, using CuKα, in the 8–145° 2θ angular range, 0.022° 2θ step size and 10 s counting time. Data were evaluated by the Rietveld method using Topas V6 (Bruker, 2016) which implements the Fundamental Parameters Approach FPA (Cheary & Coelho, 1992). Because the samples consisted of mixtures of prevailing tremolite and various amounts of phyllosilicates for which no adequate structural models exist, we decided to include exclusively tremolite in the fitting process. Tests conducted inserting also talc, chlorite and chrysotile produced instable refinement with severe correlations among unit-cell and structural parameters. As a result, this approach produced two data sets with different accuracy that will be identified in the following discussion as pure samples [i.e. those containing minor extra phase(s)] and mixture samples (i.e. those containing larger amounts of phyllosilicates).

The structure of tremolite was refined, following the procedure depicted by Ballirano et al. (2021), keeping all displacement parameters fixed to reference data (Yang & Evans, 1996) without imposing restraints on bond distances and angles. The *A* cavity was modeled imposing a single *A*(2/m) site lying at 0,½,0 to help stabilizing the refinement. Site scattering (s.s.) at *M*(1), *M*(2), *M*(3), *M*(4), and *A* was refined.

The refinement was started applying a conventional description of the isotropic peak broadening characterized by a Lorentzian (size) and a Gaussian (strain) behavior (Delhez et al., 1993). Subsequently, several models of anisotropic broadening were tested to describe the peak shape of tremolite to keep into account its fibrous morphology. The best performing one was by means of normalized symmetrized spherical harmonics function (Järvinen, 1993). Absorption effects were modelled by means of the equation of Sabine et al. (1998) for a cylindrical sample and the presence of preferred orientation was corrected using normalized symmetrized spherical harmonics functions (Järvinen, 1993). The number of terms (4th-order, eight refinable parameters) was chosen according to the procedure of Ballirano (2003) and only produced a minor improvement of the fit, coherently with extremely small parameters, as expected for a capillary mount.

### ^57^Fe Mössbauer spectroscopy

Mössbauer spectra were measured to determine the Fe^3+^ contents. Approximately 50 mg of each sample was gently crushed under acetone, then mixed with a sugar-acetone solution designed to form sugar coatings around each grain and minimize preferred orientation. Grains were heaped in a sample holder confined by Kapton polyimide film tape. Spectra were measured at room temperature (295 K) using a source of 100–60 mCi ^57^Co in Rh on a SEE Co. model WT302 spectrometer located in the laboratory of Dr. Sebastian Stoian at the University of Idaho. Run times were 24 h and spectra were collected in 2048 channels and corrected for nonlinearity.

Spectra were fitted with Lorentzian doublets using the MEX_FielDD program courtesy of Dr. Eddy DeGrave at the University of Ghent. Isomer shifts (IS, or δ), and quadrupole splittings (QS, or Δ) of all doublets were allowed to vary, and widths (full width at half maximum) of all peaks were coupled to vary in pairs except those for Fe^3+^, for which the widths were fixed.

Error bars for Mössbauer measurements are discussed at length by Dyar (1984) and Dyar et al. (2008) for fits to well-resolved spectra giving errors of ± 0.02 mm/s for δ and Δ and ± 3% absolute on areas. However, the spectra studied here are noisy due to the low Fe contents of the samples and also have multiple overlapping distributions. So the errors are likely slightly higher: ± 0.02–0.05 mm/s for IS and QS with errors of ± 3–5% absolute on areas.

To obtain true %Fe^3+^ contents, all peak areas were converted using the correction factor C = 1.22 for amphiboles as developed by Dyar et al. (1993) using comparisons between wet chemical and Mössbauer data. The equation used was:

$$\%{Fe}_{true}^{3+}=\frac{100\times \frac{1}{1.22} \times \frac{{\%Fe}_{M\ddot{o} ss}^{3+}}{100-{\%Fe}_{M\ddot{o} ss}^{3+}}}{\frac{\frac{1}{1.22} \times {\%Fe}_{M\ddot{o} ss}^{3+}}{100-{\%Fe}_{M\ddot{o} ss}^{3+}}+1}.$$

## Results and discussion

### Samples size and morphology

The combined results confirm the presence of tremolite fibres in all investigated sites (Table 2), underscoring its ubiquitous distribution within the studied area. In several samples, preliminary scrutiny of the pattern indicated the occurrence of accessory phases in the hand sample, primarily chlorite, talc, and chrysotile (Table 2), which are indicative of specific hydrothermal alteration or local metamorphic contexts.

**Table 2:** Mineralogical association of the investigated hand samples determined by XRPD, SEM-TEM-EDS, and DSC-DTG. Width of tremolite fibers as determined by TEM (average of 200 measurements for each individual sample) are also reported

Municipality	Site area	Accessory phases	Width (μm)
Castelluccio superiore	Perruttiere	Tremolite > > Chlorite and Talc	0.30
Castelluccio inferiore	Serrapollo	Tremolite > > Chlorite	0.41
Castelluccio superiore	Radicata	Tremolite > > Chlorite	0.72
Castelluccio superiore	Serra Fagosa	Tremolite > Talc > > Chlorite	0.47
Viggianello	Pastoroso	Tremolite > > Chlorite	0.23
Viggianello	Pastoroso Pidocchioso	Tremolite > Chrysotile	0.30
Viggianello^*^	Destra di Cornaleta	Tremolite > Chrysotile	0.23
Chiaromonte^*^	Cava Timpa Castello	Tremolite > Chrysotile	0.22
Lauria^*^	Tempa Bruciata	Tremolite > Chrysotile	0.31
Chiaromonte	Sagittario	Tremolite > > Chrysotile	0.19
Viggianello^*^	Falascoso	Tremolite > Chrysotile	0.16

^*^Mixture samples

**Fig. 3:**
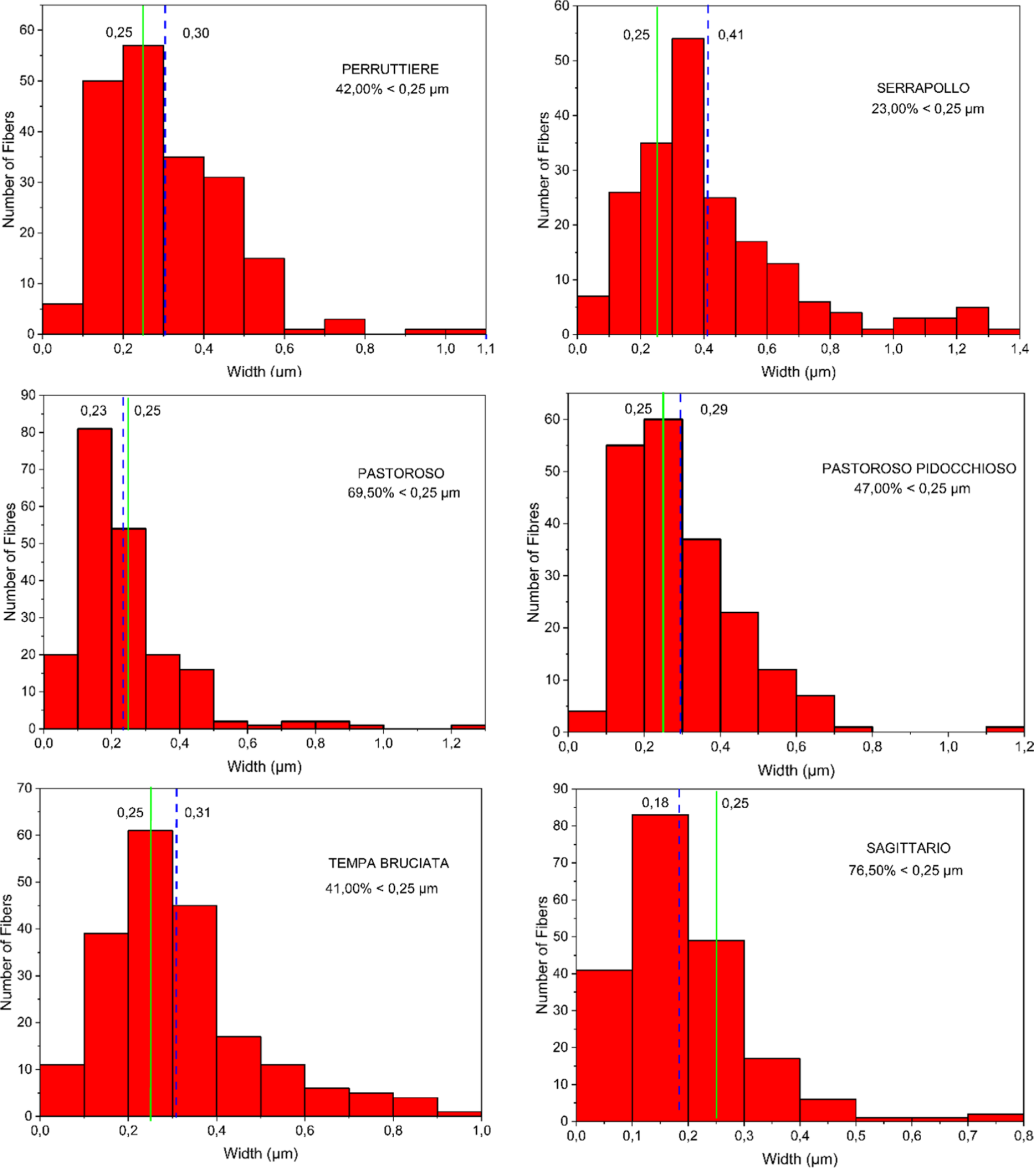

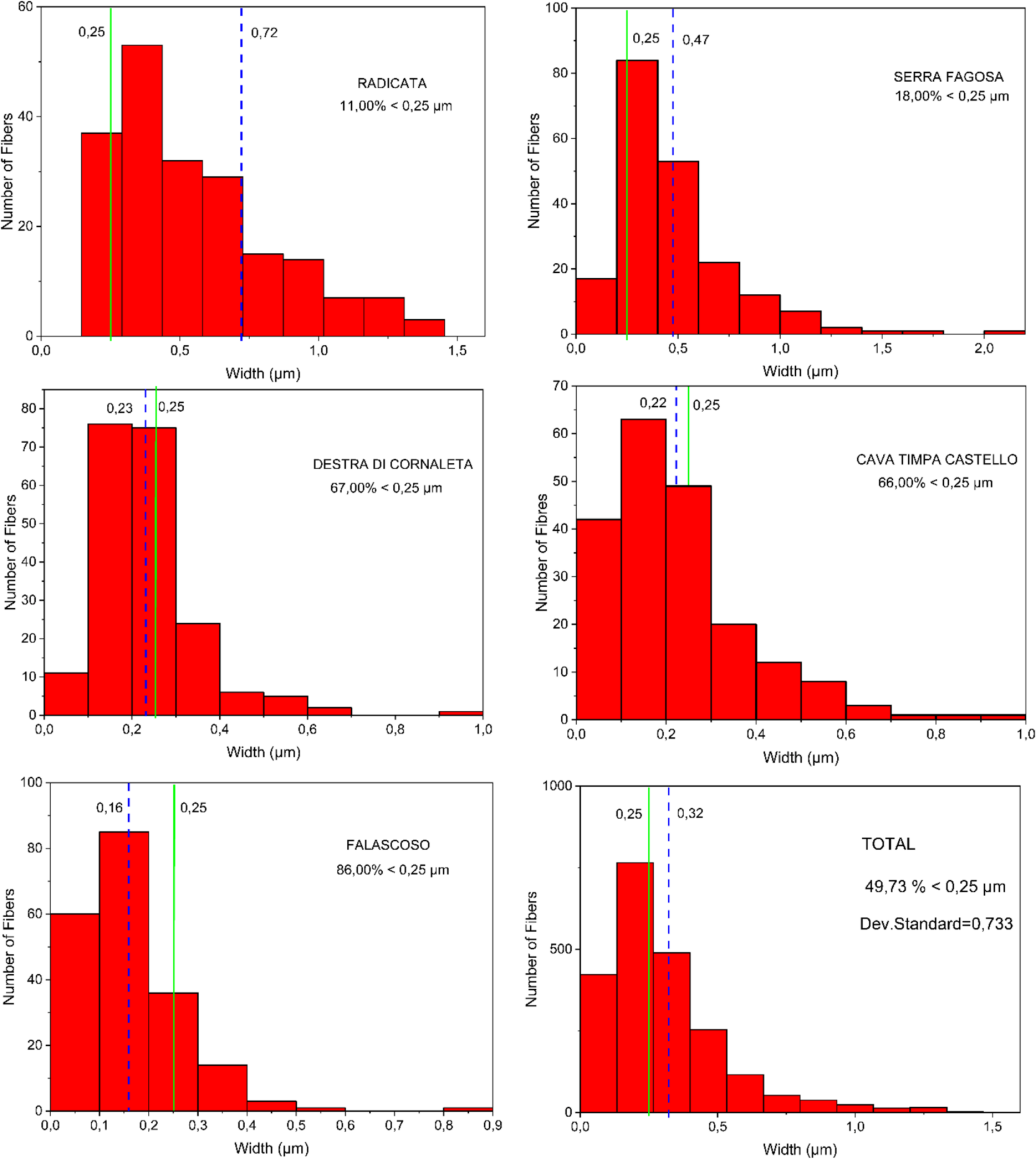
Widths of tremolite fibres, as calculated by measuring 200 single fibres for each sample. The green line at 0.25 µm can be considered as a thickness threshold. The blue line shows average value

Tremolite fibres, characterized by flexibility and lengths exceeding 5 μm (WHO, 1997), exhibited a wide distribution in diameter, with an overall mean width of 0.32 μm (Fig. 3). Analysis of 200 measurements for each of the 11 samples (Table 2) showed that approximately 50% of the fibres had a diameter of less than 0.25 µm. This finding is of particularly interest in light of recent scientific evidence of Wylie and Korchevskiy (2023) claiming that amphibole fibres longer than 5 µm with widths less than 0.25 µm should be primarily considered as potent mesothelial carcinogens, requiring special attention for public health protection. Furthermore, 98% of the analyzed fibres were found to have a diameter ≤ 1 µm (Fig. 3), conforming to the criterion proposed by Harper et al. (2012) for distinguishing between asbestos fibres and non-asbestos particles. Notably, the observed dimensional distributions confirm not only the asbestiform nature of the investigated samples but also their toxicological potential, given the high proportion of thin fibres.

### Crystal structural characterization of fibrous tremolite samples

Chemical analyses revealed substantial chemical homogeneity among the samples (Table 3). Most of the samples have an average FeO content around 2.00 wt%, except for the samples from Cava Timpa Castello and Falascoso showing a FeO content higher than 3.00 wt%, and Sagittario sample with FeO content up to ca. 4.00 wt% (Table 3).

**Table 3:** Average chemical compositions obtained by EMP analysis for the investigated fibrous tremolite samples (estimated standard deviations in brackets)

Oxides (wt%)	Perruttiere	Serrapollo	Radicata	Serra Fagosa	Pastoroso	Pastoroso Pidocchioso	Destra di Cornaleta	Cava Timpa Castello	Tempa Bruciata	Sagittario	Falascoso
SiO_2_	58.45(44)	58.63(16)	58.56(10)	58.44(44)	58.49(31)	58.57(41)	58.67(24)	58.40(14)	58.53(18)	58.25(48)	58.24(25)
TiO_2_	0.05(4)	0.01(1)	0.01(1)	0.01(2)	0.01(2)	0.02(2)	0.03(3)	0.02(2)	0.01(2)	0.03(2)	0.02(2)
Al_2_O_3_	0.06(2)	0.03(3)	0.05(11)	0.07(6)	0.15(8)	0.12(5)	0.12(13)	0.13(4)	0.10(4)	0.15(4)	0.10(4)
Cr_2_O_3_	0.01(1)	0.02(1)	0.02(2)	0.01(1)	0.02(2)	0.02(2)	0.01(2)	n.d	0.01(1)	0.01(1)	0.02(2)
MgO	23.45(33)	23.72(39)	23.30(25)	23.70(37)	23.67(41)	23.37(42)	23.46(46)	22.79(25)	23.40(19)	22.50(42)	22.90(53)
CaO	12.79(27)	13.14(29)	13.27(16)	12.97(45)	12.81(33)	13.33(29)	13.41(13)	13.26(22)	13.38(20)	12.96(63)	13.26(26)
MnO	0.12(5)	0.16(8)	0.09(4)	0.16(7)	0.13(5)	0.08(4)	0.24(20)	0.12(6)	0.09(8)	0.09(3)	0.23(29)
FeO_tot_	2.18(14)	1.74(65)	2.23(41)	1.90(33)	2.10(29)	2.13(42)	2.06(67)	3.18(34)	2.08(32)	4.10(40)	2.75(77)
Na_2_O	0.46(4)	0.20(6)	0.24(14)	0.16(5)	0.22(5)	0.10(3)	0.07(8)	0.10(3)	0.13(1)	0.05(5)	0.14(2)
K_2_O	0.09(3)	0.06(3)	0.03(2)	0.01(1)	0.03(1)	0.02(1)	0.02(1)	0.03(1)	0.02(1)	0.01(1)	0.05(2)
F	0.03(4)	0.03(5)	0.03(3)	0.02(2)	0.07(5)	0.04(3)	n.d	0.05(5)	0.03(4)	0.01(1)	0.04(6)
Cl	0.03(4)	0.04(5)	n.d	0.01(1)	0.08(5)	0.02(3)	0.05(7)	0.01(1)	0.06(4)	0.04(1)	0.01(1)
H_2_O^a^	2.17	2.17	2.18	2.18	2.14	2.17	2.18	2.16	2.16	2.17	2.16
Total	99.90	99.95	99.99	99.65	99.92	99.98	100.33	100.26	100.00	100.34	99.91
Fe_2_O_3_^b^	0.41	0.62	0.37	0.25	0.26	0.24	0.43	0.49	0.37	0.59	0.46
FeO^b^	1.81	1.19	1.89	1.68	1.87	1.92	1.67	2.73	1.75	3.57	2.33

^a^Estimated from stoichiometry. ^b^Measured by Mössbauer spectroscopy

n.d.: not detected

**Fig. 4:**
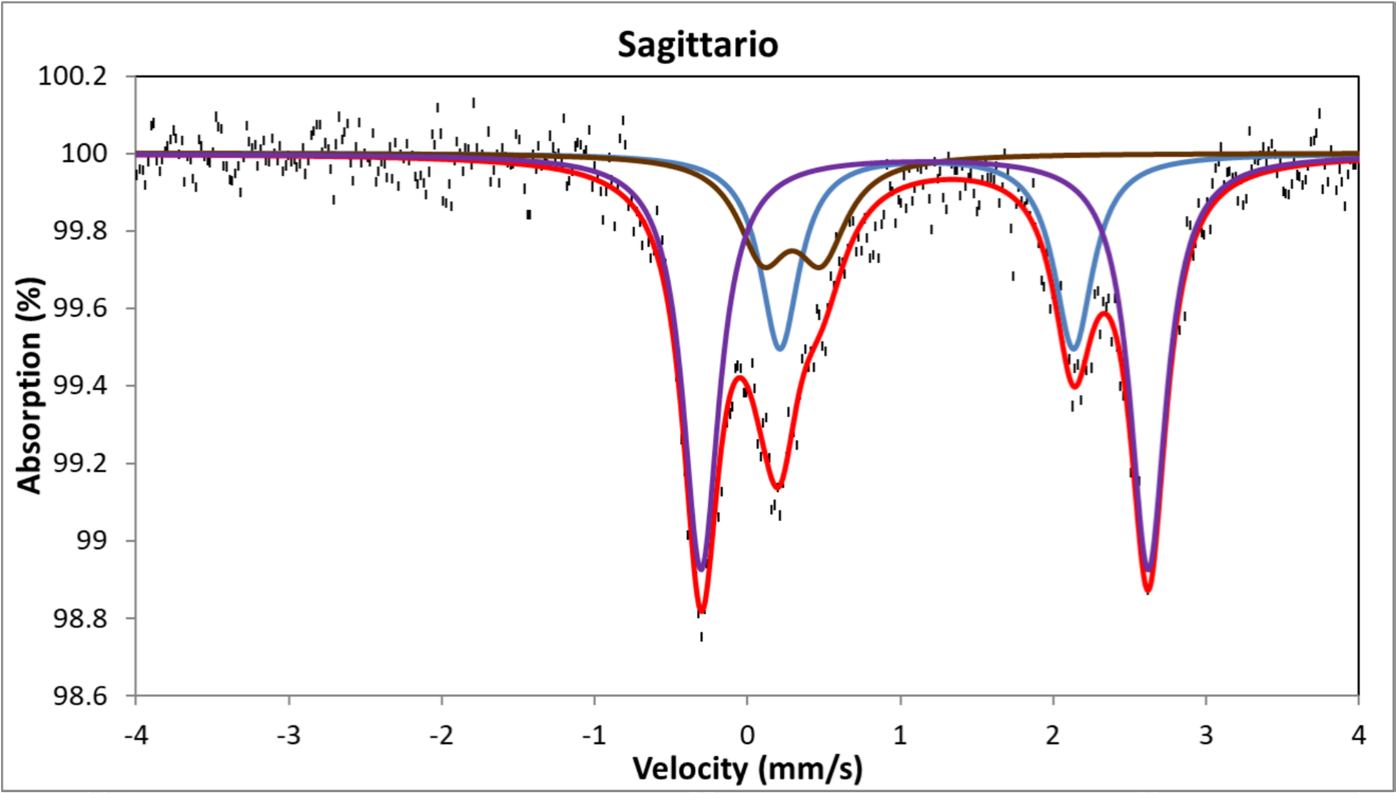
^57^Fe Mössbauer spectrum of asbestos tremolite sample (Sagittario sample) at room temperature. Fitted absorption doublets assigned to Fe^2+^ *M*(1) + *M*(3) are indicated in purple color, Fe^2+^ *M*(2) + *M*(4) in bluish color, and Fe^3+^ at *M*(2) in brown color. Dots denote measured spectrum, and red curve represents summed fitted spectrum. ^57^Fe Mössbauer spectra of all other fibrous tremolite samples are reported as supporting material (Fig. S2)

^57^Fe Mössbauer spectra (Fig. 4) are nicely reproduced by means of two well separated doublets with centroid shift at ca. 1.1 mm/s, typical for Fe^2+^, and a weak shoulder with centroid shift at ca. 0.3 mm/s, typical for Fe^3+^ (Figs. 4 and S2). It must be pointed out that, due to both the small amount of accessory phases in the hand samples and the low iron content hosted in these minerals (Table 2), their possible contribution to the Mössbauer spectrum is negligible. Table S1 reports the relevant hyperfine parameters of each spectral fitting. The Fe^2+^ contributions have quadrupole splitting of 1.9 and 2.9 mm/s, respectively, and the Fe^3+^ doublet has quadrupole splitting between 0.4 and 0.6 mm/s. The Fe^3+^ content ranged from 14 to 24% Fe_tot_ from direct area measurement for all samples (Fe_raw_), except for Serrapollo tremolite showing a Fe^3+^ content significantly higher (40% Fe_tot_). This is significantly lower than Fe^3+^ content measured for other fibrous amphiboles of environmental and health interest such as fibrous richterite from Libby (Montana, USA) and fibrous fluoro-edenite from Biancavilla (Sicily, Italy), which showed a Fe^3+^ content of 69% (Gunter et al., 2011) and ranging from 54 to 94% (Andreozzi et al., 2009), respectively. Notably, the key role of iron in inducing asbestos toxicity and pathogenicity has been known for a long time (Fubini & Mollo, 1995; IARC, 2012). Specifically, Fe^2+^ present in the crystal structure promotes the formation of highly reactive HO· species through a Fenton‐like chain reaction, inducing chronic inflammation in vivo (Gualtieri, 2017; Kamp, 2009; Toyokuni, 2009; Turci et al., 2017). Moreover, recent studies reported that the potential to generate HO· species also depends upon the fibre dissolution rate determining surface iron availability, which in turn is related to mineral crystal chemistry (Andreozzi et al., 2017).

Fe distribution among amphibole structure sites was determined following the model of Andreozzi et al. (2009): the outer doublet (quadrupole splitting of 2.9 mm/s) was attributed to the presence of Fe^2+^ at *M*(1) + *M*(3) sites, the inner doublet (quadrupole splitting of 1.9 mm/s) to Fe^2+^ at *M*(2) + *M*(4) sites, and the shoulder doublet to Fe^3+^ at *M*(2) site (Figs. 4 and S2, Table S1). In the present work for all fibrous tremolite samples roughly the 67% total Fe^2+^ was allocated at *M*(1) + *M*(3) sites, and the remaining ca. 33% was allocated at *M*(2) + *M*(4) sites. The average crystal-chemical formula of the investigated asbestos tremolite samples, retrieved by combining chemical and Mössbauer data, are reported in Table 4. All samples show Ca/ΣM very close to that of the ideal tremolite (Ca/ΣM = 0.40) and have crystal-chemical formula close to the nominal formula of tremolite, Ca_2_Mg_5_Si_8_O_22_(OH)_2_ (Table 4).

**Table 4:** Average crystal-chemical formulas of the investigated fibrous tremolite samples

Sites	Perruttiere	Serrapollo	Radicata	Serra fagosa	Pastoroso	Pastoroso pidocchioso	Destra di cornaleta	Cava timpa castello	Tempa bruciata	Sagittario	Falascoso
Si	7.99(0)	7.99(1)	7.99(0)	7.99(1)	7.98(2)	7.99(0)	7.98(1)	7.98(1)	7.99(1)	7.97(1)	7.98(1)
^IV^Al	0.01(0)	–	–	0.01(1)	0.01(2)	0.01(0)	0.02(1)	0.02(1)	0.01(0)	0.02(0)	0.02(1)
ΣT	8.00(0)	7.99(0)	7.99(0)	8.00(0)	8.00(0)	8.00(0)	8.00(0)	8.00(0)	8.00(0)	8.00(0)	8.00(0)
^VI^Al	–	–	0.01(2)	–	0.01(1)	0.01(1)	–	–	0.01(1)	–	–
Ti	–	–	–	–	–	–	–	–	–	–	–
Cr	–	–	–	–	–	–	–	–	–	–	–
Fe^3+^	0.04(0)	0.06(2)	0.04(1)	0.03(0)	0.03(0)	0.02(0)	0.04(1)	0.05(1)	0.03(1)	0.06(1)	0.05(1)
Mg	4.77(7)	4.82(7)	4.74(5)	4.83(5)	4.82(7)	4.75(6)	4.76(8)	4.64(4)	4.77(5)	4.59(6)	4.68(9)
Fe^2+^	0.19(5)	0.11(5)	0.21(4)	0.14(5)	0.14(7)	0.22(4)	0.19(6)	0.30(3)	0.19(5)	0.34(5)	0.27(8)
Σ*C*	5.00(1)	5.00(1)	5.00(1)	5.00(1)	5.00(0)	5.00(1)	5.00(0)	5.00(1)	5.00(0)	5.00(1)	4.99(1)
Mn	0.01(0)	0.02(1)	0.01(0)	0.02(1)	0.01(1)	0.01(0)	0.03(2)	0.01(1)	0.01(1)	0.01(0)	0.03(3)
Fe^2+^	0.01(4)	0.02(2)	0.01(1)	0.05(7)	0.07(6)	–	–	0.02(2)	0.01(1)	0.07(8)	–
Ca	1.88(4)	1.92(4)	1.94(2)	1.90(7)	1.87(5)	1.95(4)	1.95(2)	1.94(3)	1.96(3)	1.90(9)	1.95(4)
Na	0.10(2)	0.04(2)	0.04(2)	0.03(2)	0.04(2)	0.3(1)	0.01(2)	0.02(1)	0.02(2)	0.01(1)	0.02(1)
Σ*B*	2.00(0)	2.00(1)	2.00(1)	2.00(0)	2.00(0)	1.99(0)	2.00(1)	1.99(1)	2.00(0)	1.99(1)	2.00(0)
Na	0.02(2)	0.02(1)	0.02(2)	0.01(2)	0.02(1)	–	–	–	0.01(1)	–	0.01(1)
K	0.02(1)	0.01(1)	–	–	0.01(0)	–	–	–	–	–	0.01(0)
Σ*A*	0.04(2)	0.03(2)	0.03(2)	0.01(2)	0.02(1)	-	0.01(0)	0.01(0)	0.01(1)	-	0.02(1)
OH	1.98(2)	1.98(3)	1.98(1)	1.99(1)	1.95(3)	1.98(1)	1.98(2)	1.97(2)	1.97(2)	1.98(0)	1.98(2)
F	0.01(1)	0.01(2)	0.01(1)	0.01(1)	0.03(2)	0.02(1)	–	0.02(2)	0.01(2)	–	0.02(2)
Cl	0.01(1)	0.01(1)	–	–	0.02(1)	0.01(1)	0.01(2)	–	0.01(1)	0.01(0)	–
Σ*O*_*3*_	2.00(0)	2.00(0)	2.00(0)	2.00(0)	2.00(0)	2.00(0)	2.00(0)	2.00(0)	2.00(0)	1.99(0)	2.00(0)
Ca/ΣM^a^	0.38	0.38	0.39	0.38	0.37	0.39	0.39	0.39	0.39	0.38	0.39
X_FeA_(%)^b^	4.4	3.1	4.6	4.2	4.5	4.6	4.4	6.6	4.2	8.4	5.9

^a^M = Mg + ^VI^Fe^2+^ + Mn + Ni + Ti + Fe^3+^ + Cr^3+^ + ^VI^Al

^b^Ferro-actinolite content: X_FeA_ = (Fe^2+^ + Mn)/(Fe^2+^ + Mn + Mg)

**Fig. 5:**
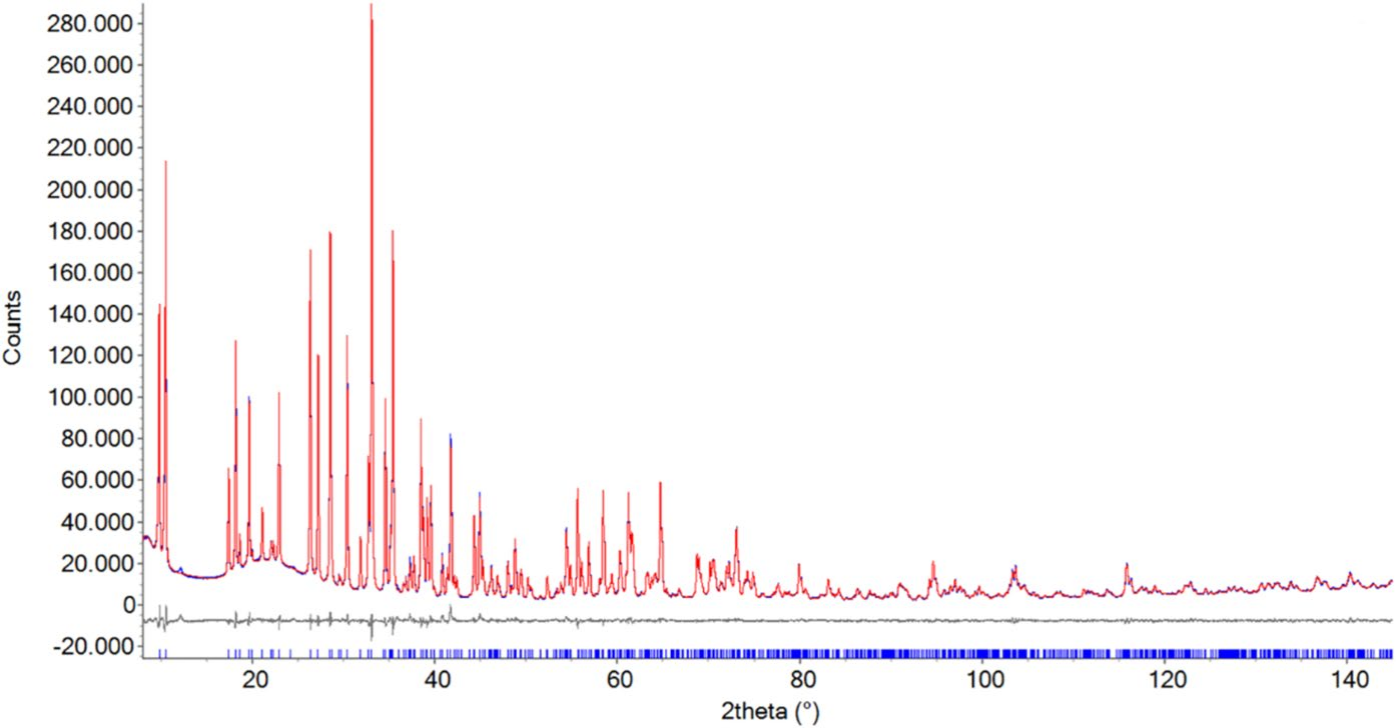
Representative Rietveld refinement plots for the sample from Castelluccio Inferiore Serrapollo. Blue dots: experimental data; red continuous line: calculated pattern; grey continuous line: difference curve; vertical blue ticks: positions of calculated Bragg reflections. The modulation of the background is due to the contribution of the borosilicate-glass capillary

**Fig. 6:**
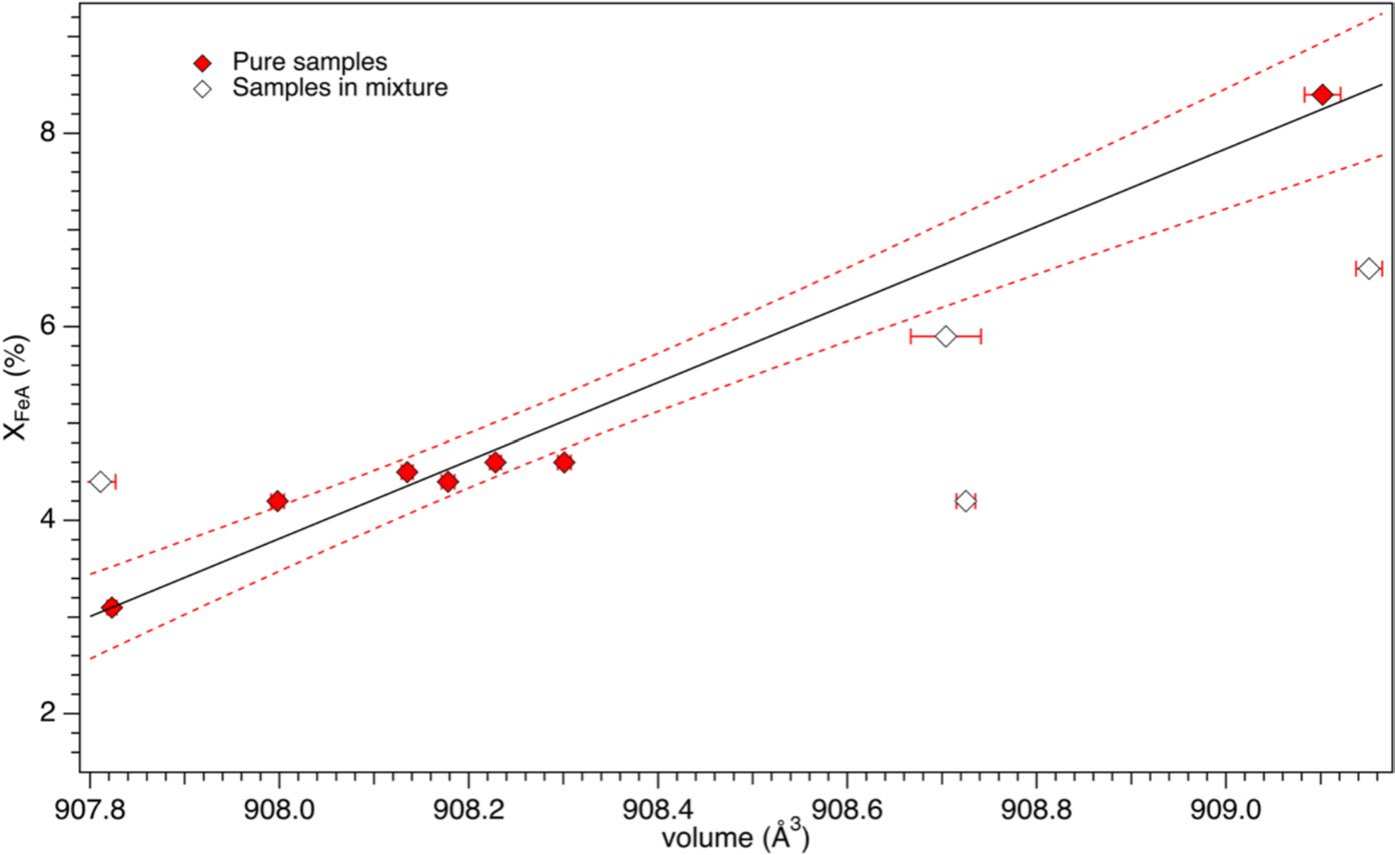
Dependency of volume from X_FeA_. Sample were subdivided into those occurring as substantially pure and those as mixture in the powders analyzed

Figure 5 shows, as an example, the conventional Rietveld plots of the refinement of the sample from Castelluccio Inferiore Serrapollo. The experimental details of the X-ray diffraction data collection, together with the refined cell parameters for all samples are reported in Tables S2 and S3, respectively. From a structural point of view, the measured cell volumes were slightly higher than that of pure tremolite (907.0 Å^3^) reported by Yang and Evans (1996). This is in agreement with the presence of ferro-actinolite content [X_FeA_ = (Fe^2+^ + Mn)/(Fe^2+^ + Mn + Mg)], ranging from approximately 3% to 8% in our samples (Table 4). The dependence of the cell volume on X_FeA_ is particularly evident if only pure samples are considered (Fig. 6). Moreover, the refined site scattering (s.s.) obtained for the C sites [*M*(1) + *M*(2) + *M*(3)] for all studied fibrous samples further confirms the presence of a scatterer cation heavier than Mg (such as Fe). The agreement between the refined s.s. and those calculated from chemical data (Table S4) is very satisfactory, with the largest difference being well below to 3% relative (the only exception is sample Cava Timpa Castello, a mixture sample, with a difference for the C sites of ca. 3.5%).

The Rietveld refinements of the pure samples indicated < T(2)-O > values ranging from 1.631 to 1.637 Å, slightly longer than the < T(1)-O > values (1.621–1.628 Å), as commonly reported for *C*2/m amphiboles with no tetrahedrally-coordinated Al (Hawthorne & Oberti, 2007).

The average bond distance at the C-sites of the pure samples ranges from 2.076 to 2.080 Å, consistent with their relatively minor chemical variability.

Thermal behavior.

**Fig. 7:**
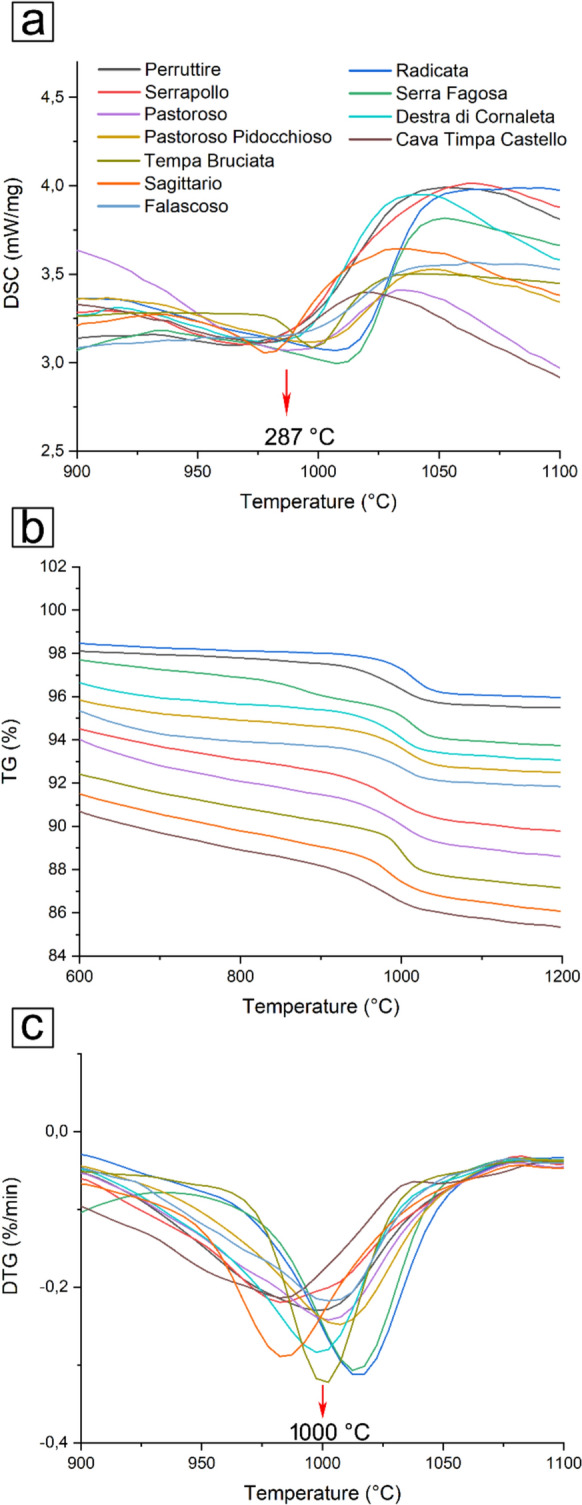
**a** DSC curves of the studied samples. The red arrow indicates the average breakdown temperature; **b** TG curves of the studied samples; **c** DTG curves of the studied samples. The red arrow indicates the average temperature of the maximum mass loss rate

The thermal behaviour of tremolite samples was investigated using DSC, TG and DTG (Fig. 7a–c). DSC analysis revealed endothermic peaks associated with structural breakdown between 964 °C and 1006 °C (987 °C average, Fig. 7a), while DTG showed dehydroxylation peaks in a closely aligned range of 982 °C to 1016 °C (Table 5; Fig. 7c). The consistency of the results obtained with these techniques confirms the tremolite breakdown event, consistent with literature data (Bloise et al., 2017b, 2018). The slight variations (of a few degrees) in the peak breakdown temperatures between individual samples are likely attributable to minor, inherent inhomogeneities within the sample material. As shown in Fig. 7a, c, the DTG peak was more readily identifiable than the DSC peak in all tremolite samples. This is because the DSC signal was more susceptible to interference from impurities and redox phenomena during the heating process (Bloise, 2023).

**Table 5:** Endothermic peak temperatures in DSC curves; peak temperatures in DTG curves; Mass loss associated with structural water dehydroxylation, derived from TG analysis. The percentage loss was calculated within the temperature range of 950–1050 °C

Phases	Perruttiere	Serrapollo	Radicata2	Serra Fagosa2	Pastoroso	Pastoroso Pidocchioso	Destra di Cornaleta	Cava Timpa Castello	Tempa Bruciata	Sagittario	Falascoso
*DSC (T °C)*											
Tremolite endo peaks	964.3	971.3	1003.4	1005.6	988.6	996.6	976.8	976.1	998.4	979.1	999.1
*DTG (T °C)*											
Tremolite	1003.7	983.6	1015.9	1015.4	1000.4	1007.2	1002.4	986.9	1000.4	982.3	1002.9
*TG loss %*											
Tremolite mass loss	2.2	2.33	2.01	2.04	2.26	2.04	2.00	2.56	2.17	2.24	2.12

Critically, all decomposition temperatures fall below the 1020 °C threshold established by Bloise (2023) for fibrous tremolite, thereby confirming this morphology in all samples.

Thermogravimetric (TG) analysis quantified the mass loss associated with the thermal decomposition of the tremolite samples. The recorded losses ranged from 2.00 to 2.56%, with an average value of approximately 2.2% (Table 5; Fig. 7b). This mass reduction is attributed to the release of structural water (dehydroxylation) during the breakdown of the tremolite structure. The measured values align closely with the theoretical water content of tremolite and are consistent with experimental data reported in the literature (Bloise, 2023), in which mass losses of around 2.0% were observed during the dehydroxylation process.

## Conclusions

This study provides the first comprehensive mineralogical, crystal-chemical, and morphological characterization of naturally occurring asbestos (NOA) tremolite from ophiolite outcrops in the Pollino area (Basilicata region, southern Italy). A multi-analytical approach (SEM-EDS, TEM-EDS, EMPA, Mössbauer spectroscopy, XRPD, and TG-DSC) was applied to eleven samples collected from sites characterized by regular human activities, including farming, construction, and quarrying. Morphological analyses revealed that tremolite fibres form bundles that easily separate into thin, respirable fibrils. Notably, about 50% of the fibres have a diameter below 0.25 µm, a size recently associated with high carcinogenic potential, and 98% are ≤ 1 µm, confirming their asbestiform nature. Thermal decomposition occurs within a narrow temperature range below 1020 °C, confirming the fibrous morphology of all samples. The fibrous tremolites are chemically homogeneous, with FeO content ranging from about 1.7 to 4.1 wt%. Fe^2+^ is preferentially allocated to *M*(1) + M(3) sites (∼ 67%) over*M*(2) + *M*(4) sites (∼ 33%), whereas Fe^3+^ is strictly confined to the *M*(2) site. Structurally, the unit-cell volumes are slightly expanded relative to pure tremolite, reflecting a minor but detectable ferro-actinolite component (3–8%). Crystal-chemical characterization showed that iron, a key factor in asbestos pathogenicity due to its ability to catalyse the formation of reactive oxygen species, is consistently present within the tremolite structure across all samples.

The identification of tremolite asbestos fibres in the collected samples raises the question of their potential impact on human health. This concern is supported by epidemiological evidence from the same geographical area (Pasetto et al., 2004). The VIII Italian National Mesothelioma Registry (ReNaM) reports 124 mesothelioma cases (1993–2021) among residents of municipalities in southern Basilicata, including Lauria, Castelluccio Superiore and Inferiore, Latronico, Episcopia, San Severino Lucano, and Francavilla in Sinni, with a predominance of pleural mesothelioma (91.9%). Although ReNaM does not define the Pollino area as a distinct epidemiological unit, municipality-level data allow a descriptive geographical comparison with the distribution of NOA-bearing lithologies. Furthermore, Caputo et al. (2018) reported a statistically significant excess of mesothelioma incidence in the Pollino area (Standardized Incidence Ratio-SIR = 208; 95% CI: 111–355), particularly in municipalities where asbestos-bearing outcrops occur close to settlements. While occupational and anthropogenic asbestos sources cannot be entirely excluded, the coexistence of NOA occurrences and an increased mesothelioma burden in the same geographical context suggests that environmental exposure deserves further investigation.

In this frame, the natural occurrence of asbestos tremolite in Basilicata region represents a potential environmental health concern, especially considering that both natural processes (e.g., landslides and slope failures) and human activities (e.g., agriculture, road works, excavations, and construction activities) may promote the release and dispersion of respirable fibres into the environment. Therefore, further multidisciplinary studies integrating geological, environmental, toxicological, and epidemiological approaches are needed to better assess exposure pathways and potential health implications. Ongoing investigations based on lung fibre burden analyses in sentinel animals may provide valuable information on environmental exposure to airborne tremolite fibres and contribute to the development of risk assessment and risk-mapping tools for the Pollino area.

## Supplementary Information

Below is the link to the electronic supplementary material.

Supplementary file1 (DOCX 10083 KB)

## Data Availability

All the raw data and material used for this research is available at the University of Calabria and Sapienza University of Rome for request.

## Declarations

### Human and animal participants

Not applicable, no human participants and/or animals were used in this research.
