# Supplementary material for: Naturally occurring asbestos and its possible environmental and health implications in the Basilicata region (Southern Italy)
Source: Environ Geochem Health. 2026 Jun 25;48(9):420. doi: 10.1007/s10653-026-03314-5 (PMC13303591; doi:10.1007/s10653-026-03314-5)
Supplement: Supplementary file 1 — Supplementary file1 (DOCX 10083 KB) [file 10653_2026_3314_MOESM1_ESM.docx]

**Naturally Occurring Asbestos and its possible Environmental and Health Implications in the Basilicata Region (Southern Italy)**

**Andrea Bloise, Paolo Ballirano, Ilaria Fuoco, Maria Cristina Di Carlo, Carmine Apollaro, Giovanni Vespasiano, Marco Paccapelo, Lorenzo Arrizza, M. Darby Dyar, Elizabeth Sklute, Alessandra Altieri, Antonella Campopiano, Annapaola Cannizzaro, Maria Rosaria Bruno, Giacomo Malvasi, and Alessandro Pacella^*^**

**Andrea Bloise, Ilaria Fuoco, Carmine Apollaroc, Giovanni Vespasiano**

Department of Biology, Ecology and Earth Sciences, University of Calabria, 87036 Arcavacata di Rende (CS), Italy

**Alessandro Pacella, Ballirano Paolo^,^ Maria Cristina Di Carlo^,^ Marco Paccapelo, Lorenzo Arrizza**

Department of Earth Sciences, Sapienza University of Rome, Piazzale Aldo Moro, 5-I- 00185 Roma, Italy

**M. Darby Dyar, Elizabeth Sklute**

Planetary Science Institute, 1700 East Fort Lowell, Suite 106, Tucson, AZ, USA

**Antonella Campopiano, Annapaola Cannizzaro, Alessandra Altieri**

Department of Medicine, Epidemiology, Occupational and Environmental Hygiene, National Institute for Insurance against Accidents at Work (INAIL), Rome, Italy

**Maria Rosaria Bruno**

Department of Medicine, Epidemiology, Occupational and Environmental Hygiene, National Institute for Insurance against Accidents at Work (INAIL), Lamezia Terme, Italy

**Giacomo Malvasi**

Radioactivity and Asbestos Office ARPA Basilicata, Potenza, Italy

Corresponding author:

**Alessandro Pacella**

Department of Earth Sciences, Sapienza University of Rome, Piazzale Aldo Moro, 5-I- 00185 Roma, Italy

^*^e-mail address: [alessandro.pacella@uniroma1.it](mailto:alessandro.pacella@uniroma1.it)


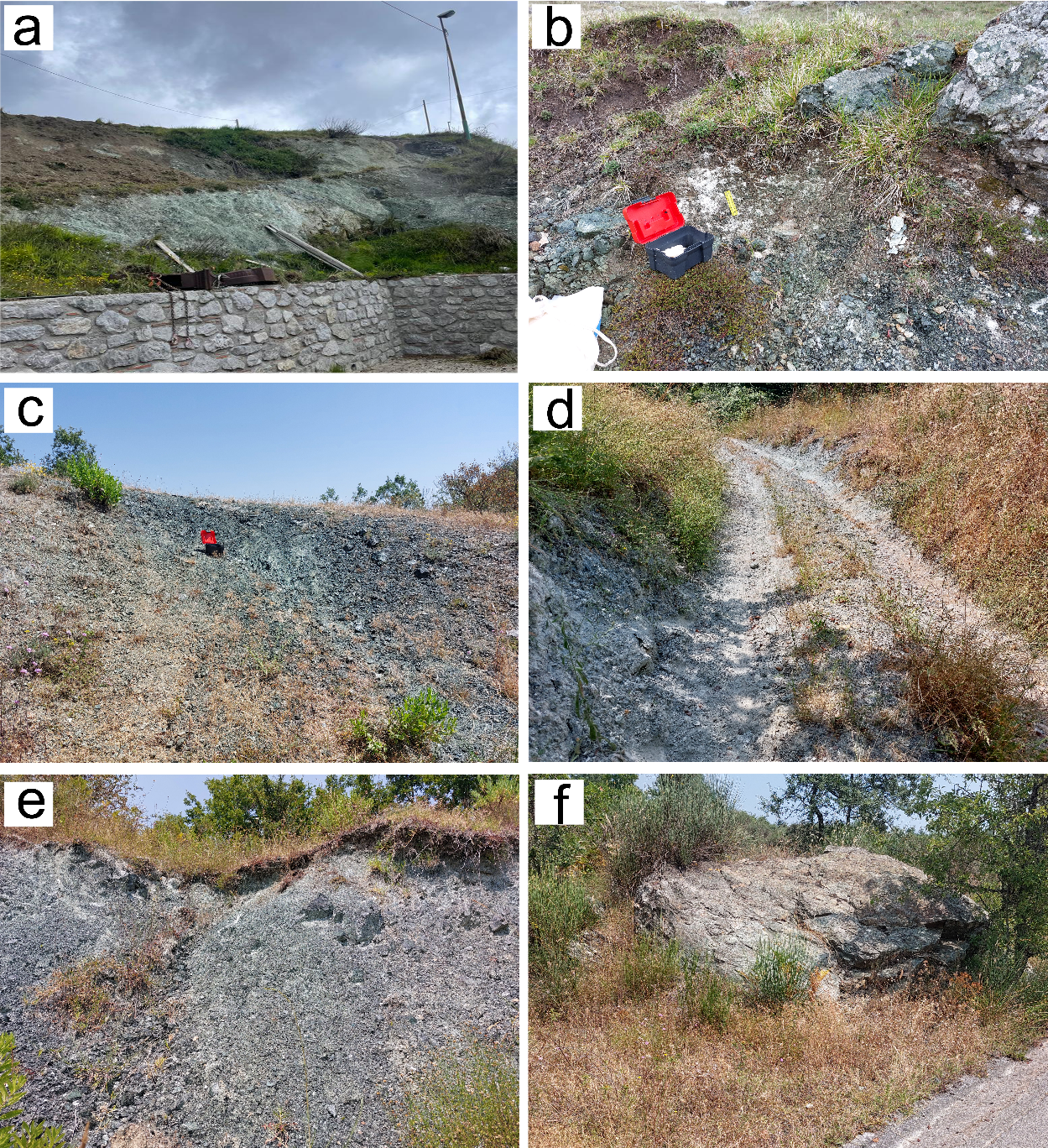


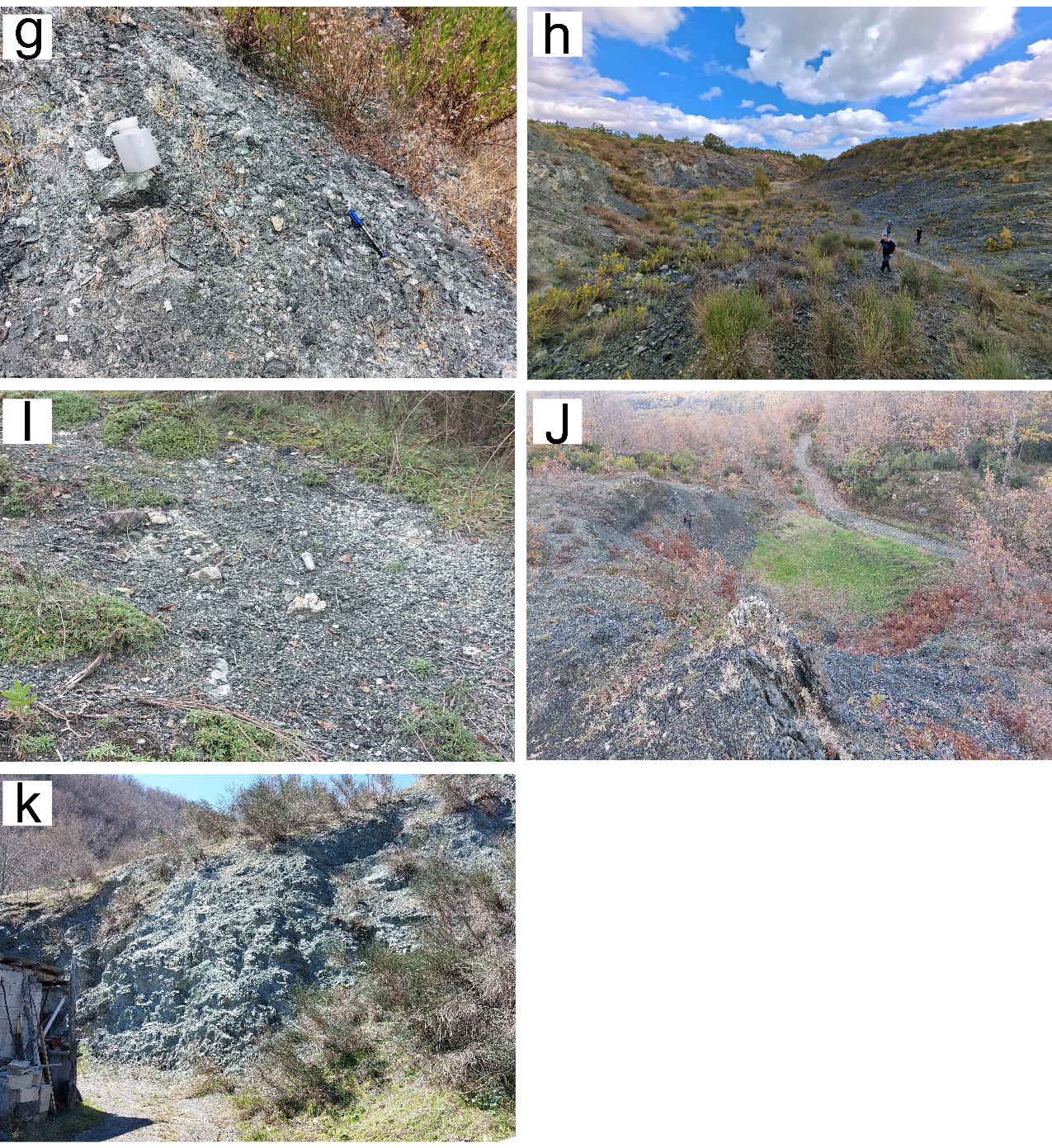


**Fig. S1** Pictures taken at the site samples (Basilicata region, Italy), **a)** Perruttiere, excavation for house construction; **b)** Serrapollo, road cutting; **c**) Radicata, outcrop; **d**) Serra Fagosa, dirt road; **e**) Pastoroso, excavation front; **f**) Pastoroso Pidocchioso, outcrop; **g)** Destra di Cornaleta, landslide; **h)** Cava Timpa, Castello, inactive quarry; **i)** Tempa Bruciata, landslide slope; **j)** Sagittario, excavation front; **k)** Falascoso, excavation front.


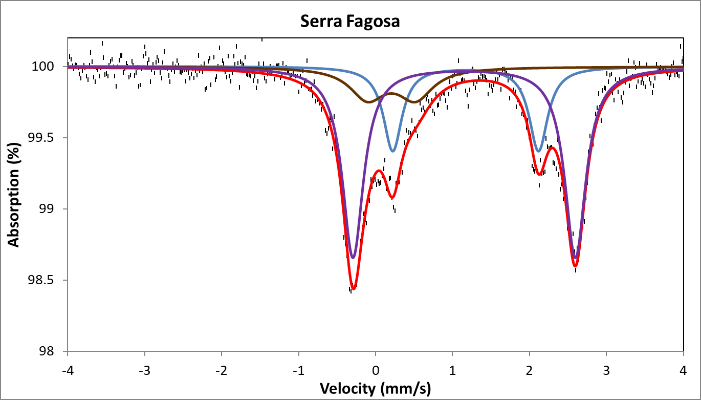

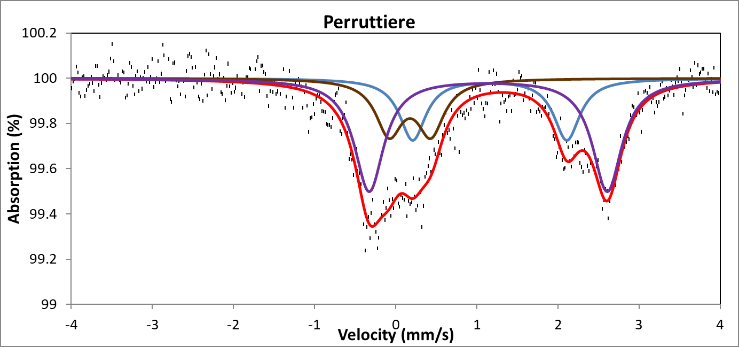

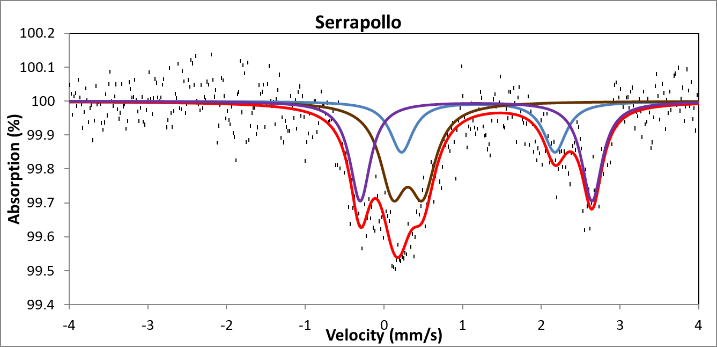


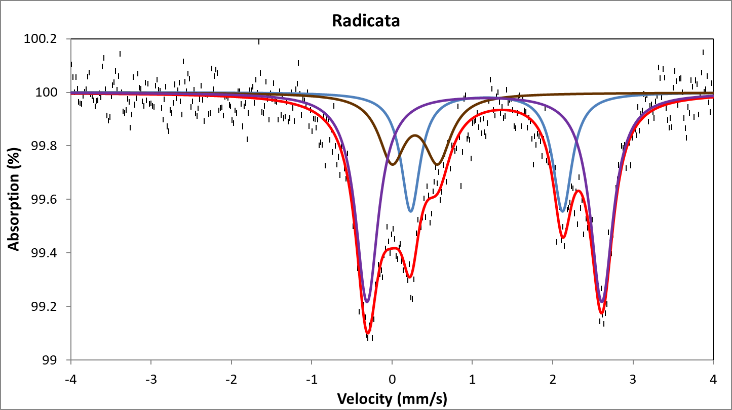


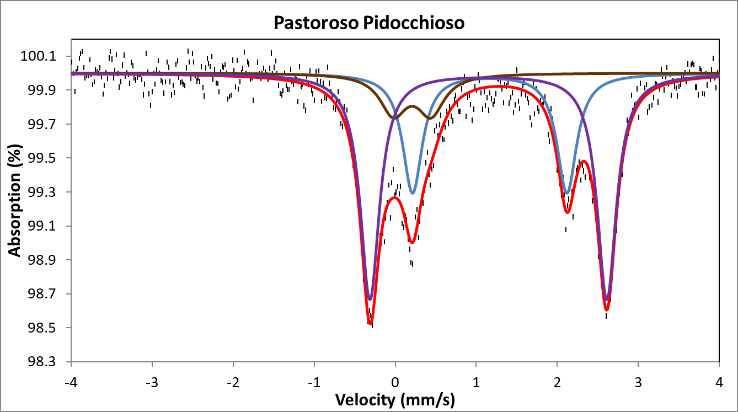

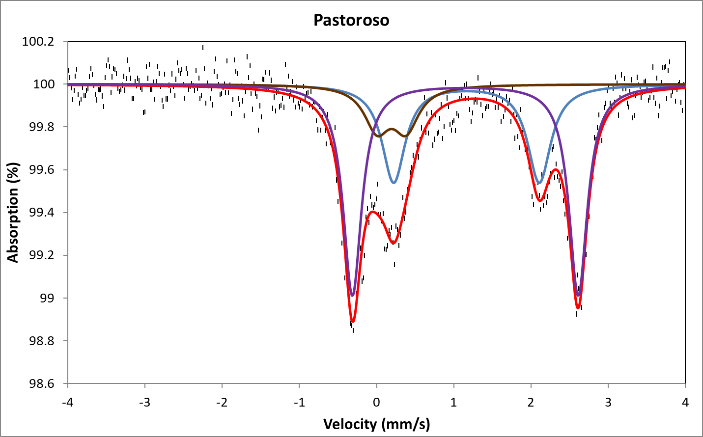


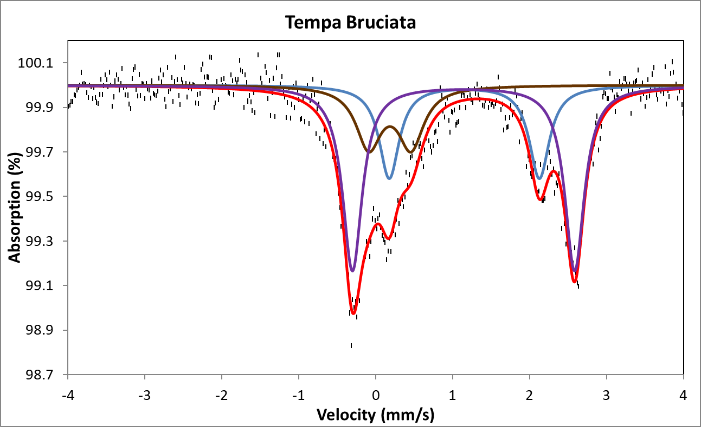

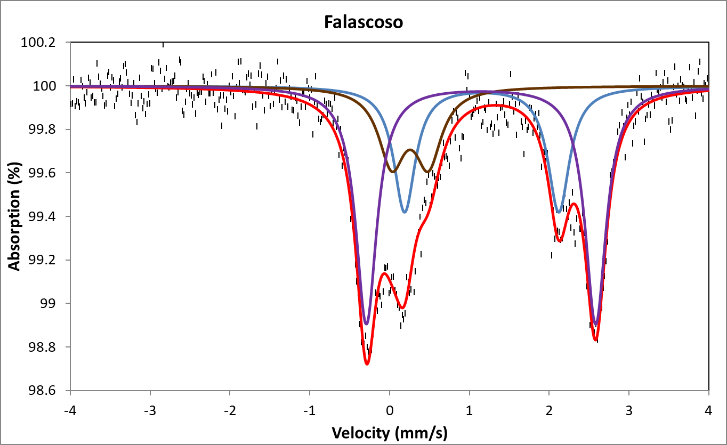

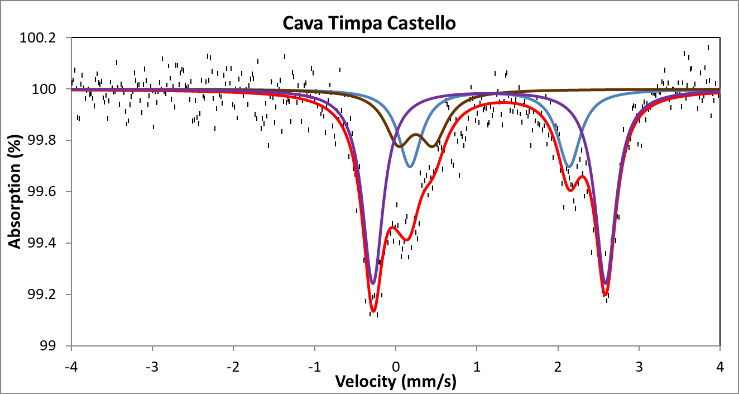

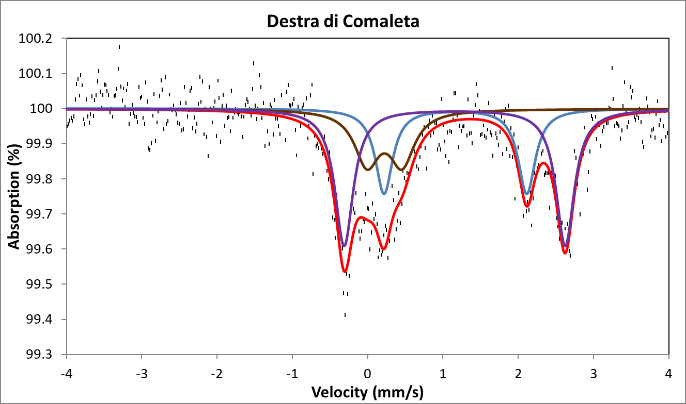


**Fig. S2** ^57^Fe Mössbauer spectrum of asbestos tremolite sample at room temperature. Fitted absorption doublets assigned to Fe^2+^ M(1) + M(3) are indicated in purple color, Fe^2+^ M(2) + M(4) in bluish color, and Fe^3+^ at M(2) in brown color. Dots denote measured spectrum, and red curve represents summed fitted spectrum.

Table S1. Mössbauer parameters for the investigated fibrous tremolite samples measured at room temperature. *δ* = centroid shift (mm/s), Δ*E_Q_* = quadrupole splitting (mm/s), Γ = full width at half maximum (mm/s).

| Sample | *δ* | *ΔE_Q_* | Γ | % Area | Assignment |
| --- | --- | --- | --- | --- | --- |
| Perruttiere | 1.14 | 2.94 | 0.44 | 53 | Fe^2+^ *M*(1) + *M*(3) |
|  | 1.16 | 1.90 | 0.39 | 25 | Fe^2+^ *M*(2) + *M*(4) |
|  | 0.17 | 0.51 | 0.40^*^ | 22 | Fe^3+^ *M*(2) |
| Serra Pollo | 1.17 | 2.95 | 0.32 | 38 | Fe^2+^ *M*(1) + *M*(3) |
|  | 1.20 | 1.95 | 0.35 | 22 | Fe^2+^ *M*(2) + *M*(4) |
|  | 0.30 | 0.38 | 0.40^*^ | 40 | Fe^3+^ *M*(2) |
| Radicata | 1.15 | 2.92 | 0.35 | 55 | Fe^2+^ *M*(1) + *M*(3) |
|  | 1.18 | 1.89 | 0.29 | 25 | Fe^2+^ *M*(2) + *M*(4) |
|  | 0.28 | 0.57 | 0.40^*^ | 20 | Fe^3+^ *M*(2) |
| Serra Fagosa | 1.15 | 2.89 | 0.33 | 60 | Fe^2+^ *M*(1) + *M*(3) |
|  | 1.17 | 1.89 | 0.29 | 24 | Fe^2+^ *M*(2) + *M*(4) |
|  | 0.21 | 0.62 | 0.55 | 16 | Fe^3+^ *M*(2) |
| Pastoroso | 1.15 | 2.92 | 0.29 | 54 | Fe^2+^ *M*(1) + *M*(3) |
|  | 1.16 | 1.89 | 0.37 | 31 | Fe^2+^ *M*(2) + *M*(4) |
|  | 0.19 | 0.38 | 0.40^*^ | 15 | Fe^3+^ *M*(2) |
| Pastoroso Pidocchioso | 1.15 | 2.92 | 0.29 | 56 | Fe^2+^ *M*(1) + *M*(3) |
|  | 1.17 | 1.91 | 0.30 | 30 | Fe^2+^ *M*(2) + *M*(4) |
|  | 0.21 | 0.47 | 0.40^*^ | 14 | Fe^3+^ *M*(2) |
| Destra di Cornaleta | 1.16 | 2.94 | 0.30 | 48 | Fe^2+^ *M*(1) + *M*(3) |
|  | 1.17 | 1.90 | 0.28 | 28 | Fe^2+^ *M*(2) + *M*(4) |
|  | 0.22 | 0.47 | 0.40^*^ | 24 | Fe^3+^ *M*(2) |
| Cava Timpa Castello | 1.15 | 2.87 | 0.31 | 57 | Fe^2+^ *M*(1) + *M*(3) |
|  | 1.16 | 1.96 | 0.35 | 25 | Fe^2+^ *M*(2) + *M*(4) |
|  | 0.25 | 0.43 | 0.40^*^ | 18 | Fe^3+^ *M*(2) |
| Tempa Bruciata | 1.14 | 2.89 | 0.31 | 52 | Fe^2+^ *M*(1) + *M*(3) |
|  | 1.16 | 1.94 | 0.32 | 28 | Fe^2+^ *M*(2) + *M*(4) |
|  | 0.22 | 0.60 | 0.40^*^ | 20 | Fe^3+^ *M*(2) |
| Sagittario | 1.16 | 2.93 | 0.29 | 55 | Fe^2+^ *M*(1) + *M*(3) |
|  | 1.17 | 1.92 | 0.31 | 28 | Fe^2+^ *M*(2) + *M*(4) |
|  | 0.29 | 0.38 | 0.40^*^ | 17 | Fe^3+^ *M*(2) |
| Falascoso | 1.15 | 2.87 | 0.32 | 52 | Fe^2+^ *M*(1) + *M*(3) |
|  | 1.16 | 1.93 | 0.33 | 28 | Fe^2+^ *M*(2) + *M*(4) |
|  | 0.26 | 0.46 | 0.40^*^ | 20 | Fe^3+^ *M*(2) |

^*^ Kept fixed throughout the refinement

Table S2 Experimental details of the X-ray diffraction data collection.

| Instrument |  | Bruker AXS D8 Advance | |  |  |
| --- | --- | --- | --- | --- | --- |
| X-ray tube |  | CuKα at 40 kV and 40 mA | | | |
| Incident beam optic | | Focussing Multilayer X-ray mirrors | |  |  |
| Sample mount |  | Rotating capillary (60 rpm) | | | |
| Soller slits |  | 2 (2.3° divergence + radial) | | | |
| Divergence slits | | 0.6 mm |  |  |  |
| Detector |  | Position Sensitive Detector (PSD) VÅNTEC-1 opening window 6° 2θ. | | | |
|  |  |  |  |  |  |
| 2θ range (°) |  | 8-145 | |  |  |
| Step size (°) |  | 0.0218 | |  |  |
| Counting time (s) |  | 10 | |  |  |

Table S3 Unit-cell parameters of fibrous tremolite samples. Reference data of prismatic tremolite, Y&E96 (Yang and Evans, 1996) are shown for comparison.

|  | ***a* (*Å*)** | ***b* (*Å*)** | ***c* (*Å*)** | ***β(°)*** | ***V* (*Å*^3^)** |
| --- | --- | --- | --- | --- | --- |
|  |  |  |  |  |  |
| **Perruttiere** | 9.84786(4) | 18.05972(7) | 5.27978(2) | 104.7225(3) | 908.178(7) |
| **Serrapollo** | 9.84756(3) | 18.05977(6) | 5.27856(1) | 104.7510(2) | 907.823(5) |
| **Radicata** | 9.84854(4) | 18.06180(8) | 5.27945(2) | 104.7365(3) | 908.228(6) |
| **Serra Fagosa** | 9.84607(5) | 18.06249(8) | 5.27930(2) | 104.7397(3) | 907.998(7) |
| **Pastoroso** | 9.84752(4) | 18.06218(7) | 5.27938(2) | 104.7384(3) | 908.135(6) |
| **Pastoroso Pidocchioso** | 9.84815(4) | 18.06408(8) | 5.27972(2) | 104.7491(3) | 908.301(7) |
| **Destra di Cornaleta** | 9.84677(1) | 18.06204(18) | 5.27843(5) | 104.7584(7) | 907.811(16) |
| **Cava Timpa Castello** | 9.85337(9) | 18.07026(16) | 5.28025(4) | 104.7574(7) | 909.151(14) |
| **Tempa Bruciata** | 9.85058(7) | 18.06762(12) | 5.27992(3) | 104.7523(5) | 908.725(10) |
| **Sagittario** | 9.85052(11) | 18.0716(2) | 5.28074(7) | 104.7431(10) | 909.102(19) |
| **Falascoso** | 9.8512(2) | 18.0717(4) | 5.27857(11) | 104.7659(18) | 908.70(4) |
| **Y&E96** | 9.8356(12) | 18.0557(22) | 5.2785(6) | 104.782(9) | 906.4(2) |
|  | | | | | |

Table S4 Site scattering (s.s.) values in electrons per formula unit for the investigated fibrous tremolite samples, obtained from the structure refinement (left) and calculated from the assigned site population (right).

|  | s.s. |  |  |  |  | s.s. |
| --- | --- | --- | --- | --- | --- | --- |
| **Perruttiere** | from |  | Assigned site population | | | from assigned |
|  | refinement |  |  |  |  | site population |
| *M*(1) | 24.47(5) |  | Mg_1.91_; Fe^2+^_0.09_ | | | 25.26 |
| *M*(2) | 24.77(5) |  | Mg_1.90_; Fe^3+^_0.04_; Fe^2+^_0.06_ | | | 25.40 |
| *M*(3) | 12.75(4) |  | Mg_0.96_; Fe^2+^_0.04_ | | | 12.56 |
| *C* site sum | **61.99(14)** |  |  |  |  | **63.22** |
| *M*(4) | 39.31(6) |  | Ca_1.88_; Na_0.10_; Fe^2+^_0.01_; Mn_0.01_ | | | 39.21 |
| *B* site sum | **39.31(6)** |  |  | | | **39.21** |
| *A* (2/m) | **0.83(4)** |  | K_0.02_; Na_0.02_ | | | **0.60** |
|  |  |  |  |  |  |  |
|  | s.s. |  |  |  |  | s.s. |
| **Serrapollo** | from |  | Assigned site population | | | from assigned |
|  | refinement |  |  |  |  | site population |
| *M*(1) | 24.32(3) |  | Mg_1.94_; Fe^2+^_0.06_ | | | 24.96 |
| *M*(2) | 24.31(4) |  | Mg_1.91_; Fe^3+^_0.06_; Fe^2+^_0.03_ | | | 25.26 |
| *M*(3) | 12.54(3) |  | Mg_0.97_; Fe^2+^_0.03_ | | | 12.33 |
| *C* site sum | **61.17(10)** |  |  |  |  | **62.55** |
| *M*(4) | 39.74(4) |  | Ca_1.92_; Na_0.05_; Fe^2+^_0.01_; Mn_0.02_ | | | 39.71 |
| *B* site sum | **39.74(4)** |  |  | | | **39.71** |
| *A* (2/m) | **0.65(3)** |  | K_0.01_; Na_0.01_ | | | **0.30** |
|  |  |  |  |  |  |  |
|  | s.s. |  |  |  |  | s.s. |
| **Radicata** | from |  | Assigned site population | | | from assigned |
|  | refinement |  |  |  |  | site population |
| *M*(1) | 24.77(5) |  | Mg_1.89_; Fe^2+^_0.11_ | | | 25.54 |
| *M*(2) | 24.57(5) |  | Mg_1.90_; Fe^3+^_0.04_; Fe^2+^_0.05_; Al_0.01_ | | | 25.27 |
| *M*(3) | 12.80(4) |  | Mg_0.95_; Fe^2+^_0.05_ | | | 12.70 |
| *C* site sum | **62.14(13)** |  |  |  |  | **63.51** |
| *M*(4) | 39.48(6) |  | Ca_1.94_; Na_0.04_; Fe^2+^_0.01_; Mn_0.01_ | | | 39.75 |
| *B* site sum | **39.48(6)** |  |  | | | **39.75** |
| *A* (2/m) | **0.87(3)** | Na_0.02_ | | | | **0.22** |
|  |  |  |  |  |  |  |
|  | s.s. |  |  |  |  | s.s. |
| **Serra Fagosa** | from |  | Assigned site population | | | from assigned |
|  | refinement |  |  |  |  | site population |
| *M*(1) | 24.54(6) |  | Mg_1.92_; Fe^2+^_0.08_ | | | 25.12 |
| *M*(2) | 24.38(6) |  | Mg_1.95_; Fe^2+^_0.02_; Fe^3+^_0.03_ | | | 24.70 |
| *M*(3) | 12.39(5) |  | Mg_0.96_; Fe^2+^_0.04_ | | | 12.56 |
| *C* site sum | **61.31(17)** |  |  |  |  | **62.38** |
| *M*(4) | 40.27(8) |  | Ca_1.90_; Na_0.03_; Fe^2+^_0.05_; Mn_0.02_ | | | 40.13 |
| *B* site sum | **40.27(8)** |  |  | | | **40.13** |
| *A* (2/m) | **0.38(4)** |  | Na_0.01_ | | | **0.11** |
|  |  |  |  |  |  |  |
|  | s.s. |  |  |  |  | s.s. |
| **Pastoroso** | from |  | Assigned site population | | | from assigned |
|  | refinement |  |  |  |  | site population |
| *M*(1) | 24.82(5) |  | Mg_1.90_; Fe^2+^_0.10_ | | | 25.40 |
| *M*(2) | 24.47(5) |  | Mg_1.97_; Fe^3+^_0.03_; Al_0.01_ | | | 24.55 |
| *M*(3) | 12.77(4) |  | Mg_0.95_; Fe^2+^_0.05_ | | | 12.70 |
| *C* site sum | **62.06(13)** |  |  |  |  | **62.65** |
| *M*(4) | 39.60(6) |  | Ca_1.87_; Na_0.06_; Fe^2+^_0.06_; Mn_0.01_ | | | 39.87 |
| *B* site sum | **39.60(6)** |  |  | | | **39.87** |
| *A* (2/m) | **0.72(3)** |  | K_0.01_ | | | **0.19** |
|  |  |  |  |  |  |  |
|  | s.s. |  |  |  |  | s.s. |
| **Pastoroso Pidocchioso** | from |  | Assigned site population | | | from assigned |
|  | refinement |  |  |  |  | site population |
| *M*(1) | 24.90(5) |  | Mg_1.90_; Fe^2+^_0.10_ | | | 25.40 |
| *M*(2) | 24.42(5) |  | Mg_1.90_; Fe^3+^_0.02_; Fe^2+^_0.07_; Al_0.01_ | | | 25.27 |
| *M*(3) | 12.95(3) |  | Mg_0.95_; Fe^2+^_0.05_ | | | 12.70 |
| *C* site sum | **62.28(14)** |  |  |  |  | **63.37** |
| *M*(4) | 39.71(6) |  | Ca_1.95_ Na_0.03_; Mn_0.01_ | | | 39.58 |
| *B* site sum | **39.71(6)** |  |  | | | **39.58** |
| *A* (2/m) | **0.85(4)** |  |  | | | **-** |
|  |  |  |  |  |  |  |
|  | s.s. |  |  |  |  | s.s. |
| **Destra di Cornaleta** | from |  | Assigned site population | | | from assigned |
|  | refinement |  |  |  |  | site population |
| *M*(1) | 25.02(10) |  | Mg_1.92_; Fe^2+^_0.08_ | | | 25.12 |
| *M*(2) | 24.28(11) |  | Mg_1.88_; Fe^3+^_0.04_; Fe^2+^_0.07_ | | | 25.42 |
| *M*(3) | 13.02(8) |  | Mg_0.96_; Fe^2+^_0.04_ | | | 12.56 |
| *C* site sum | **62.32(29)** |  |  |  |  | **63.10** |
| *M*(4) | 40.53(13) |  | Ca_1.95_; Na_0.01_; Mn_0.03_ | | | 39.86 |
| *B* site sum | **40.53(13)** |  |  | | | **39.86** |
| *A* (2/m) | **1.13(8)** |  | | | | **-** |
|  |  |  |  |  |  |  |
|  | s.s. |  |  |  |  | s.s. |
| **Cava Timpa Castello** | from |  | Assigned site population | | | from assigned |
|  | refinement |  |  |  |  | site population |
| *M*(1) | 25.39(9) |  | Mg_1.86_; Fe^2+^_0.14_ | | | 25.96 |
| *M*(2) | 24.01(9) |  | Mg_1.85_; Fe^3+^_0.05_; Fe^2+^_0.10_ | | | 26.10 |
| *M*(3) | 13.32(7) |  | Mg_0.93_; Fe^2+^_0.07_ | | | 12.98 |
| *C* site sum | **62.73(24)** |  |  |  |  | **65.04** |
| *M*(4) | 39.82(11) |  | Ca_1.94_; Na_0.02_; Fe^2+^_0.01_; Mn_0.01_ | | | 39.53 |
| *B* site sum | **39.82(11)** |  |  | | | **39.53** |
| *A* (2/m) | **0.93(6)** |  |  | | | **-** |

|  | s.s. |  |  |  |  | s.s. |
| --- | --- | --- | --- | --- | --- | --- |
| **Tempa Bruciata** | From |  | Assigned site population | | | from assigned |
|  | refinement |  |  |  |  | site population |
| *M*(1) | 25.27(7) |  | Mg_1.92_; Fe^2+^_0.08_ | | | 25.12 |
| *M*(2) | 24.71(7) |  | Mg_1.89_; Fe^3+^_0.03_; Fe^2+^_0.07_; Al_0.01_ | | | 24.41 |
| *M*(3) | 12.94(6) |  | Mg_0.96_; Fe^2+^_0.04_ | | | 12.92 |
| *C* site sum | **62.91(20)** |  |  |  |  | **62.45** |
| *M*(4) | 40.05(9) |  | Ca_1.96_; Na_0.02_; Fe^2+^_0.01_; Mn_0.01_ | | | 39.93 |
| *B* site sum | **40.05(9)** |  |  | | | **39.93** |
| *A* (2/m) | **0.68(5)** |  | Na_0.01_ | | | **0.11** |

|  | s.s. |  |  |  |  | s.s. |
| --- | --- | --- | --- | --- | --- | --- |
| **Sagittario** | From |  | Assigned site population | | | from assigned |
|  | refinement |  |  |  |  | site population |
| *M*(1) | 26.02(15) |  | Mg_1.82_; Fe^2+^_0.18_ | | | 26.52 |
| *M*(2) | 24.89(15) |  | Mg_1.86_; Fe^3+^_0.06_; Fe^2+^_0.08_ | | | 25.96 |
| *M*(3) | 13.33(11) |  | Mg_0.91_; Fe^2+^_0.09_ | | | 13.26 |
| *C* site sum | **64.23(41)** |  |  |  |  | **65.74** |
| *M*(4) | 39.62(17) |  | Ca_1.90_; Na_0.01_; Fe^2+^_0.06_; Mn_0.01_ | | | 39.92 |
| *B* site sum | **39.63(17)** |  |  | | | **39.92** |
| *A* (2/m) | **0.79(10)** |  |  | | | **-** |

|  | s.s. |  |  |  |  | s.s. |
| --- | --- | --- | --- | --- | --- | --- |
| **Falascoso** | From |  | Assigned site population | | | from assigned |
|  | refinement |  |  |  |  | site population |
| *M*(1) | 26.47(21) |  | Mg_1.88_; Fe^2+^_0.12_ | | | 25.68 |
| *M*(2) | 23.52(23) |  | Mg_1.86_; Fe^3+^_0.05_; Fe^2+^_0.09_ | | | 25.96 |
| *M*(3) | 13.36(17) |  | Mg_0.94_; Fe^2+^_0.06_ | | | 12.84 |
| *C* site sum | **63.35(61)** |  |  |  |  | **64.84** |
| *M*(4) | 39.99(27) |  | Ca_1.95_; Na_0.02_; Mn_0.03_ | | | 39.97 |
| *B* site sum | **39.99(27)** |  |  | | | **39.97** |
| *A* (2/m) | **2.70(17)** |  | K_0.01_; Na_0.01_ | | | **0.30** |
